# Supplementary material for: Local electronic descriptors for solute-defect interactions in bcc refractory metals
Source: Nat Commun. 2019 Oct 2;10:4484. doi: 10.1038/s41467-019-12452-7 (PMC6775119; doi:10.1038/s41467-019-12452-7)
Supplement: Supplementary file 1 — Supplementary Information [file 41467_2019_12452_MOESM1_ESM.pdf]

Supplementary Information

Local electronic descriptors for solute-defect interactions in bcc refractory metals

Hu et al.

Section Index

Supplementary Method....3

Supplementary Note 1....11

Supplementary Note 2....13

Supplementary Note 3....20

Supplementary Note 4....22

Supplementary Note 5....25

Supplementary Note 6....28

Supplementary Note 7....30

Supplementary Note 8....32

Supplementary Note 9....35

Supplementary Note 10....44

Table Index

Supplementary Table 1. 3
Supplementary Table 2.. 13
Supplementary Table 3. 16
Supplementary Table 4. 21
Supplementary Table 5. 21
Supplementary Table 6.. 22
Supplementary Table 7. 40

Figure Index

Supplementary Figure 1....4
Supplementary Figure 2.....5
Supplementary Figure 3.....7
Supplementary Figure 4.....8
Supplementary Figure 5.....9
Supplementary Figure 6....10
Supplementary Figure 7.....11
Supplementary Figure 8.....12
Supplementary Figure 9....23
Supplementary Figure 10. 23
Supplementary Figure 11.....24
Supplementary Figure 12.....25
Supplementary Figure 13.....26
Supplementary Figure 14.....30
Supplementary Figure 15. 33
Supplementary Figure 16.....39
Supplementary Figure 17.....42
Supplementary Figure 18.....43

Supplementary Method

Electronic configurations of the pseudopotentials for DFT calculations

Supplementary Table 1. The electronic configuration of the pseudopotential for each element used in the first-principles calculations. The electrons in the bracket are treated as inner-core electrons.

Elements	Hf_pv	Ta_pv	W_pv
V_RHFIN	*([Xe]4f^{14})5p^{6}5d^{3}6s^{1}*	*([Xe]4f^{14})5p^{6}5d^{4}6s^{1}*	*([Xe]4f^{14})5p^{6}5d^{5}6s^{1}*
Elements	Re	Os	Ir
V_RHFIN	*([Xe]4f^{14})5d^{6}6s^{1}*	*([Xe]4f^{14})5d^{7}6s^{1}*	*([Xe]4f^{14})5d^{8}6s^{1}*
Elements	Pt		
V_RHFIN	*([Xe]4f^{14})5d^{9}6s^{1}*		

Atomistic geometries of the supercells used for first-principles calculations

In the following paragraphs, the input size and geometry of the simulation supercells used for the first-principles calculations are described in detail along with its k-point sampling.

Vacancy: A bcc 4×4×4 supercell is used to model the electronic structures of the mono-vacancy defect and the corresponding solute-vacancy interactions in bcc Ta and W. One vacancy per supercell is introduced by removing a single Ta or W atom, as shown in Supplementary Figure 1a. The atomic sites chosen for the solute occupation are labeled by numbers in Supplementary Figure 1a. A larger number, n, means a longer pairing distance between the solute atom and the defect center. The atomic site labeled with "*ref*" serves as the reference site in perfect bcc lattice for calculating the solute-defect interaction energy. A 3×3×3 grid is used for the k-point mesh in the first-principles calculations.

Self-interstitial atom defects (SIA): In the present work, two types of high symmetry SIA defects are studied, namely the <100>- and <111>-dumbbell structures. The initial input supercell geometries of the two defects are shown in Supplementary Figures 1b and 1c, respectively. The solute-substitutional sites are also labeled following the same notation as for the vacancy-solute interactions. Based on the supercell size (a bcc 4×4×4 supercell), a 3×3×3 grid is used for the k-point mesh in the first-principles calculations. Moreover, after relaxation, it is found that the <111>-dumbbell structure is actually transformed to the <111>-crowdion structure, which is consistent with the previous calculation results[1].

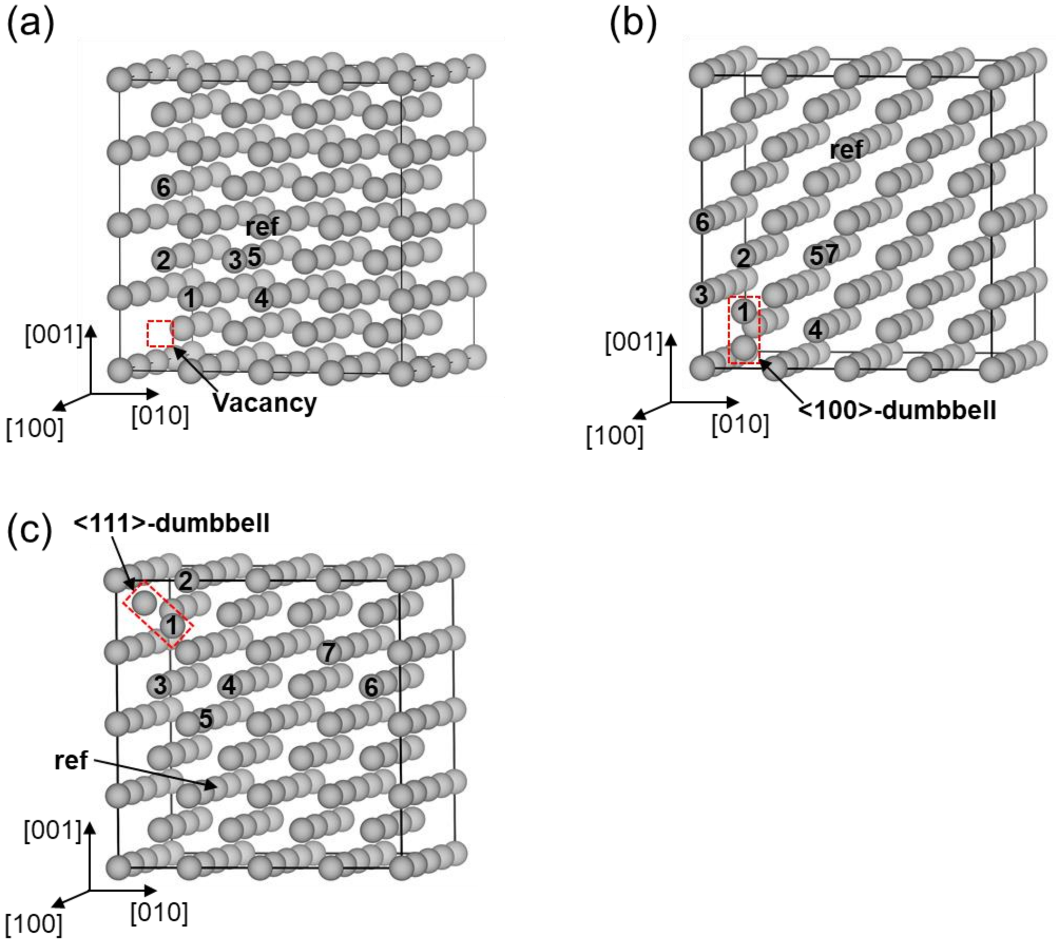

Supplementary Figure 1. The input configurations of the supercells for the point defects. (a) Vacancy; (b) <100>-dumbbell; (c) <111>-dumbbell. The atomic sites for solute occupation are marked by numbers according to their relative distance to the defect center.

$\frac{1}{2}\langle 111 \rangle$ **screw dislocation:** The supercell constructed for calculating the relaxed structure of the $\frac{1}{2}\langle 111 \rangle$ screw dislocation has a geometry related to the bcc lattice index in terms of $5[11\bar{2}] \times 9[\bar{1}10] \times \frac{1}{2}[111]$. A single dislocation is placed in the center of the supercell, marked as a red dot in Supplementary Figure 2. The initial geometry of the dislocation is obtained by displacing the atoms according to the integral formulation of the anisotropic elastic displacement field[2]. The supercell contains 270 atoms and has a repeat length of one Burgers vector along the dislocation line direction. The supercell is relaxed via first-principles calculations using the flexible boundary condition method[3,4] with a k-point mesh of 1×1×16. The relaxation scheme is detailed described in our previous publication[5]. The relaxed supercell is then doubled along the z-axis (dislocation line direction) to make a new supercell. The latter is used for the calculations of solute-dislocation interactions. The purpose of this is to minimize the potential solute-solute interactions from the image interactions due to periodic boundary conditions in the DFT calculations.

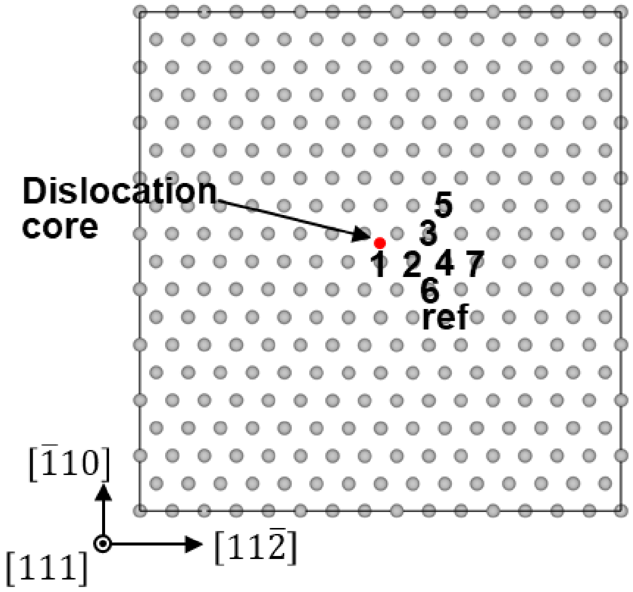

Supplementary Figure 2. The input configurations of the supercell used for the calculations of the $\frac{1}{2}[111]$ screw dislocation. The atomic sites for solute occupation are marked by numbers according to their relative distance to the dislocation core.

Twin/grain boundaries with low-index interface: In the present work, first-principle calculations are performed for one twin boundary (TB) and three types of symmetric

grain boundaries (GB) (i.e. Σ3 $(11\bar{2})$ TB, Σ3 (111) GB, Σ5 (310) GB and Σ5 (210) GB). The atomic configurations of these four interfacial defects are constructed using the coincidence site lattice (CSL) model. The input geometries of the supercells are shown in Supplementary Figures 3a, 3b, 3c and 3d for the Σ3 $(11\bar{2})$ TB, Σ3 (111) GB, Σ5 (310) GB and Σ5 (210) GB, respectively. As shown in Supplementary Figures 3, two interfaces are included in one supercell due to the periodic boundary conditions and are located apart from each other by around 20 Å to avoid the image interactions. The sizes of the supercells in the bcc lattice index are $6[11\bar{2}] \times [111] \times [1\bar{1}0]$, $7[111] \times [1\bar{1}0] \times [11\bar{2}]$, $4[310] \times [\bar{1}30] \times [001]$ and $6[210] \times [\bar{1}20] \times 2[001]$ for the Σ3 $(11\bar{2})$ TB, Σ3 (111) GB, Σ5 (310) GB and Σ5 (210) GB, respectively. Based on the supercell size, the grid of the k-point mesh for the first-principles calculations is set as 1×7×9, 1×9×5, 1×4×5 and 1×6×13. Similar to Supplementary Figures 1 and 2, the atomic sites chosen for the solute occupation are also labeled in Supplementary Figure 3. For each substitutional configuration, only one solute atom was introduced, giving an in-plane solute concentration of 50 at.%. Furthermore, it is noteworthy that the Σ5 (210) GB are found to lose its mirror symmetry after the structural relaxation. The similar phenomena were also reported in the previous works[6]. The relaxed structures of the Σ5 (210) GB in pure W and Ta configurations are shown in Supplementary Figures 3e and 3f, respectively.

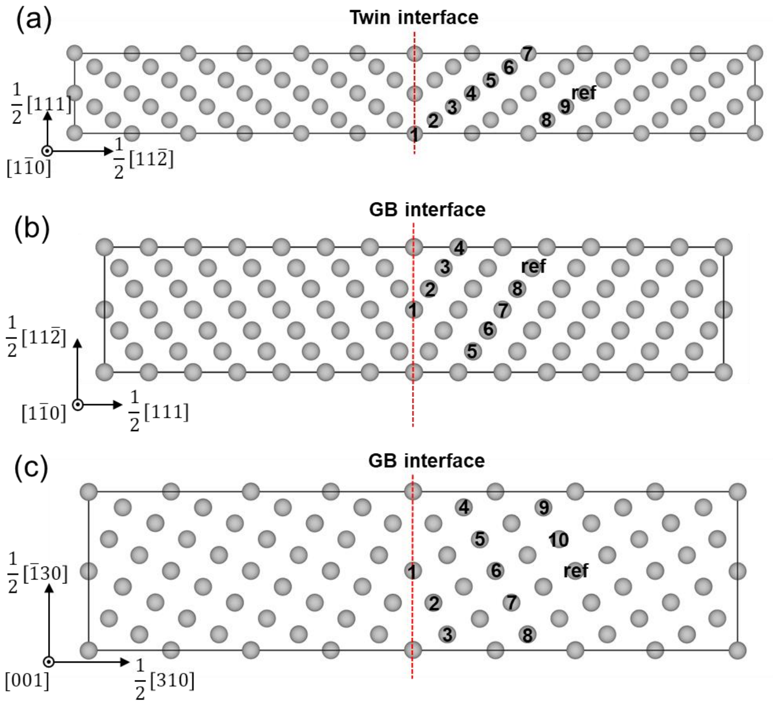

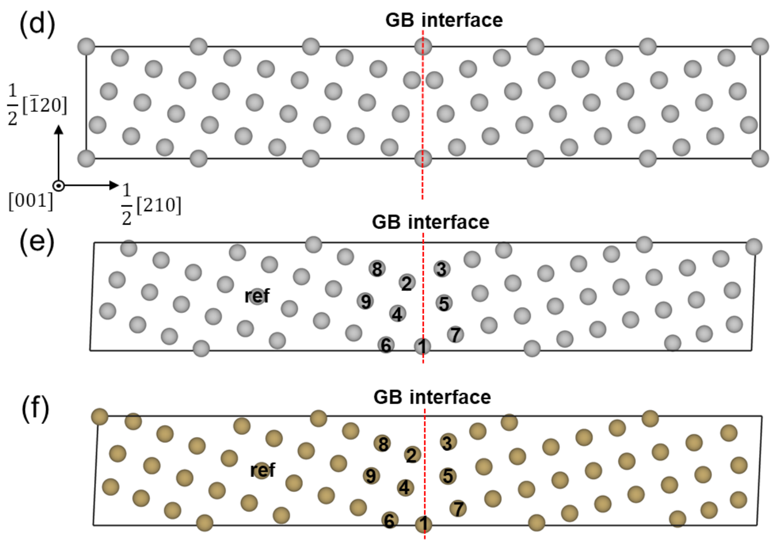

Supplementary Figure 3. Configurations of the supercells for the TB and GBs. (a) Input geometry of the Σ3 $(11\bar{2})$ TB; (b) Input geometry of the Σ3 (111) GB; (c) Input geometry of the Σ5 (310) GB; (d) Input geometry of the Σ5 (210) GB; (e) Optimized geometry of the Σ5 (210) GB in pure W; (e) Optimized geometry of the Σ5 (210) GB in pure Ta. The atomic sites for solute occupation are marked by numbers according to their relative distance to the interface.

Complex GB structures with high-index interface: Additionally, we also performed DFT calculations on two complex GB structures with low-index GB plane, namely Σ13 (230)-GB and Σ27 (552)-GB, which are used as a test data set to validate the predictions from the regression model established based on the data of other DFT calculations. The sizes of the supercells used for DFT calculations in the bcc index are $[\bar{3}20] \times 2[230] \times 2[001]$ (208 atoms) and $2[1\bar{1}0] \times \frac{1}{4}[\bar{1}\bar{1}5] \times 5.61[552]$ (with 15 Å vacuum, 237 atoms) for the Σ13 (230)-GB and Σ27 (552)-GB, respectively. Correspondingly, the k-point grid for the first-principles calculations is set to 3×1×6 and 2×2×1 (5×4×1 for the electronic structure calculation of the Σ27 (552)-GB). It is noteworthy that the input geometry of the Σ27 (552)-GB is not constructed using the coincidence site lattice (CSL) theory but implemented from a ground state structure in W predicted by a state-of-art evolutionary structure search algorithm[7,8]. Since this GB structure does not have the minor symmetry with the GB as the mirror plane, 15 Å of vacuum was applied along the GB plane normal direction to prevent the formation of two different GB structures in one supercell. For the sake of a clear visualization, in addition to marking them by numbers, the atomic sites chosen for the solute substitution are also marked in red color while the reference site is marked in blue color. The atomistic configurations of the W

supercells after relaxation are shown in Supplementary Figures 4a and 4b for the Σ13 (230)-GB and Σ27 (552)-GB, respectively. In addition, because the corresponding calculations are quite computationally consuming, the solute-defect interaction energies are only calculated for partial defect sites. So 11 sites for the Σ13 (230)-GB and 15 sites for the Σ27 (552)-GB were used for all the five solute-matrix element pairs investigated here. More sites that are very close to the GB-plane were also included for the specific W-Pt pair in the Σ27 (552)-GB case, and the results show no noticeable differences in the prediction accuracy of the solute segregation energies/concentrations when more sites were used.

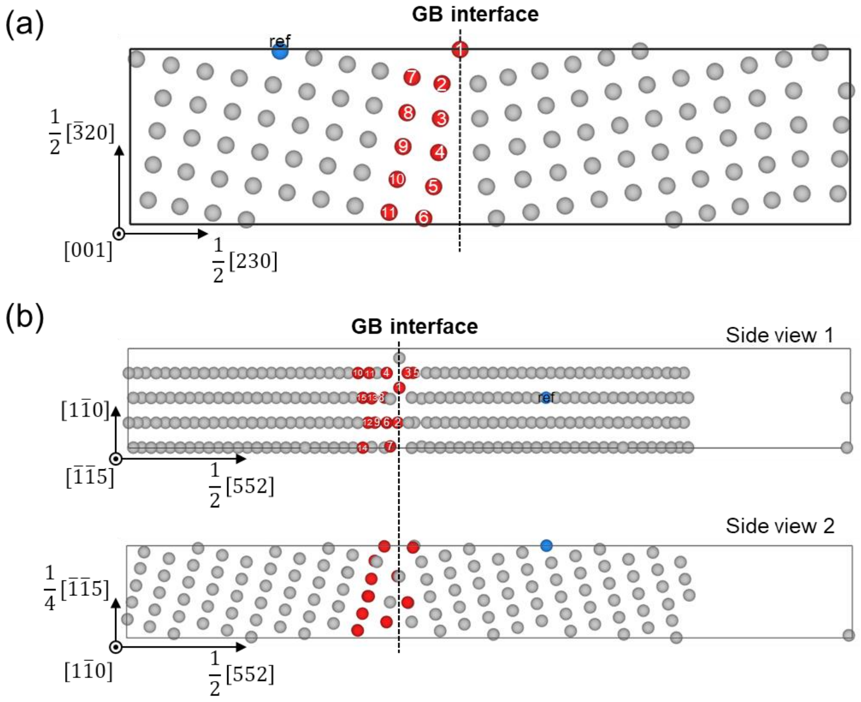

Supplementary Figure 4. The atomistic configurations of the W supercells after relaxation. (a) Σ13 (230)-GB (208 atoms); (b) Σ27 (552)-GB (237 atoms). The atomic sites chosen for the solute substitution are marked in red color while the reference site is marked in blue color. The numbers on atoms correspond to the site number in Supplementary Tables 2 and 3.

Generalized stacking faults (GSF): In the present work, the GSF of two typical slip systems in bcc crystal, namely <111>{110} and <111>{112}, are modeled via first-principles calculations. The supercell of the $[111](1\bar{1}0)$ GSF is constructed with the periodicity in the slip plane by setting the x-axis along the [110] and the y-axis along [001] directions; along the z-axis there are 16 layers of the $(1\bar{1}0)$ planes with two atoms

per plane, plus 12 Å of vacuum. Similarly, the supercell of the $[111](11\bar{2})$ GSF is composed of 12 layers of the $(11\bar{2})$ planes, plus 12 Å of vacuum. The x-axis and y-axis of the supercell are along the [111] and $[1\bar{1}0]$ directions, respectively. During GSF calculations, a slip vector $\vec{u}$ is applied by shifting the top half supercells rigidly along the [111] direction. Under the slip vector, first-principles calculations are performed by relaxing all atoms only along the z-axis. The atomic configurations of the GSF supercells before applying slip are shown in Supplementary Figures 5. Based on the supercell geometries, the k-mesh points are set as 9×13×1 and 9×15×1for the calculations of the $[111](1\bar{1}0)$ GSF and $[111](11\bar{2})$ GSF, respectively. The solute-defect interaction is only studied for the atomic site exactly on the fault plane. The solute-occupied defect and the reference site are both marked out in Supplementary Figures 5. One solute is introduced to substitute one solvent atom in the relaxed GSF supercell, which gives an in-plane solute concentration of 50 at.%. In addition, the electronic structures of the GSF of the entire $(1\bar{1}0)$ plane, and its corresponding interactions with Re solute are modeled using the same supercell as it for the calculations of the $[111](1\bar{1}0)$ GSF. The $(1\bar{1}0)$ plane is screened by shifting the top half supercells rigidly along both the [001] and $[1\bar{1}0]$ directions.

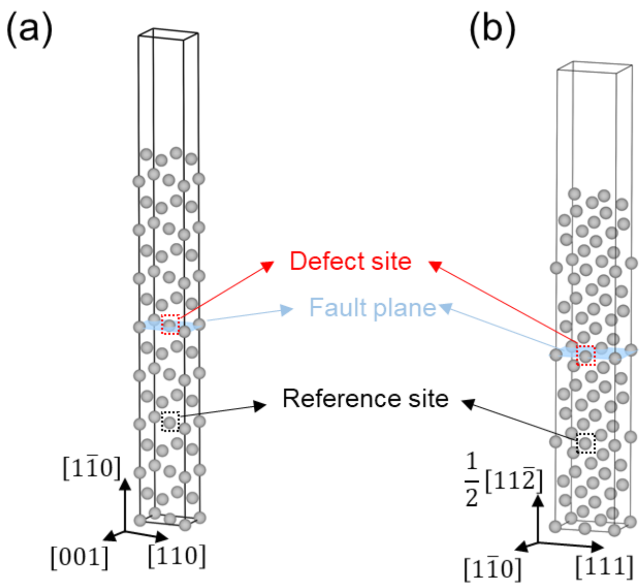

Supplementary Figure 5. Configuration of the supercells used for the calculations of GSFs before applying slip. (a) $[111](1\bar{1}0)$ GSF; (b) $[111](11\bar{2})$ GSF. The atomic sites occupied by the solute atoms are marked out.

Supplementary Figure 6

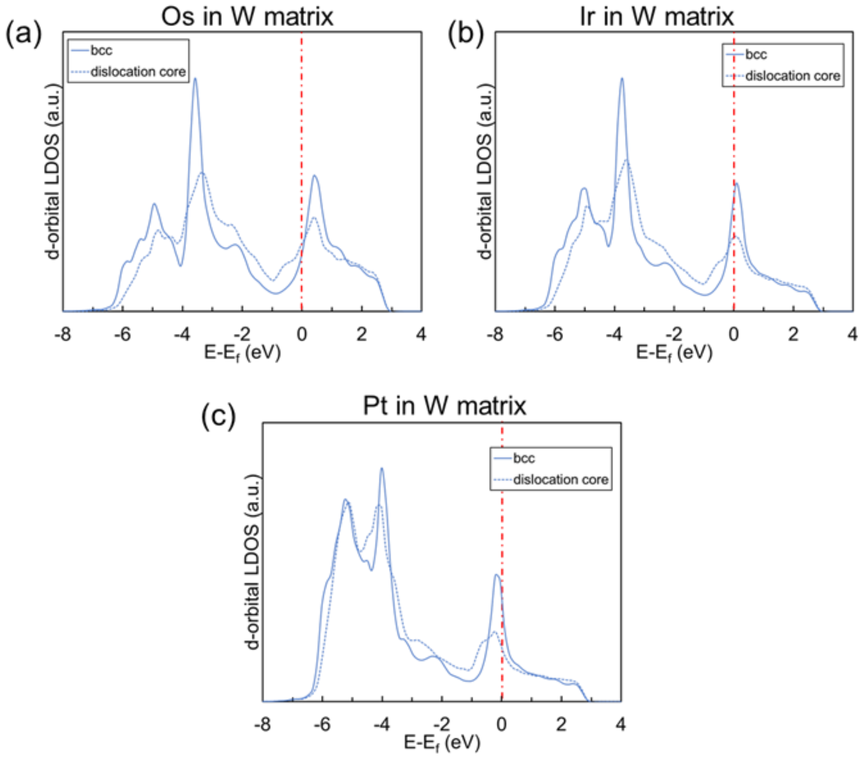

Supplementary Figure 6. *d*-orbital LDOSs of the solute atoms in the $\frac{1}{2}\langle 111\rangle$ screw dislocation W. (a)-(c) LDOS of an Os, Ir and Pt atom occupying the bcc bulk site (solid-blue line) and the $\frac{1}{2}\langle 111\rangle$ dislocation core site (dashed-blue line) in the W matrix, respectively. The bcc bulk site and dislocation core site refer to the atomic sites marked in blue and red color in Fig. 1a in the main text, respectively.

Supplementary Note 1

Sensitivities of the Δdip calculations to DFT settings

It is necessary to test the sensitivities of the Δdip measurements to the DFT parameters related to LDOS calculations, such as the number of bins of the LDOS, *k*-point density, cutoff energy for the plane-wave basis and width of the Methfessel-Paxton smearing. For the sake of simplification, the test calculations were performed for several conventional bcc supercells in which an W atom was moved away from its equilibrium position as shown in Supplementary Figure 7. As a result, the atom with a larger perturbation should have a more significant variation in general in terms of the LDOS bimodality compared with the W atom in the perfect bcc lattice. To calculate the Δdip parameter of the perturbated atom, a W atom in the unperturbed bcc cell is used as the reference. For each of the test, we only vary the value of one corresponding DFT parameter and keep others the same as those used for the defect calculations in the main text.

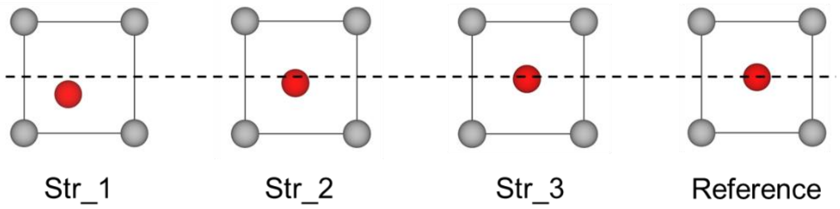

Supplementary Figure 7. Configurations of the supercells used for the calculations to test the sensitivities of the Δdip measurements to the LDOS-related DFT parameters. In Str_1, Str_2 and Str_3, the W atom perturbed from its original equilibrium position is marked in red color. The LDOS of the red atom is used to calculate the Δdip parameter.

The total number of bins of the LDOS is varied from 100 to 2000 to evaluate the corresponding effects on the Δdip measurements. As shown in Supplementary Figure 8, the values of the Δdip parameter for all three tested structures become converged when the total number of bins increases over 200.

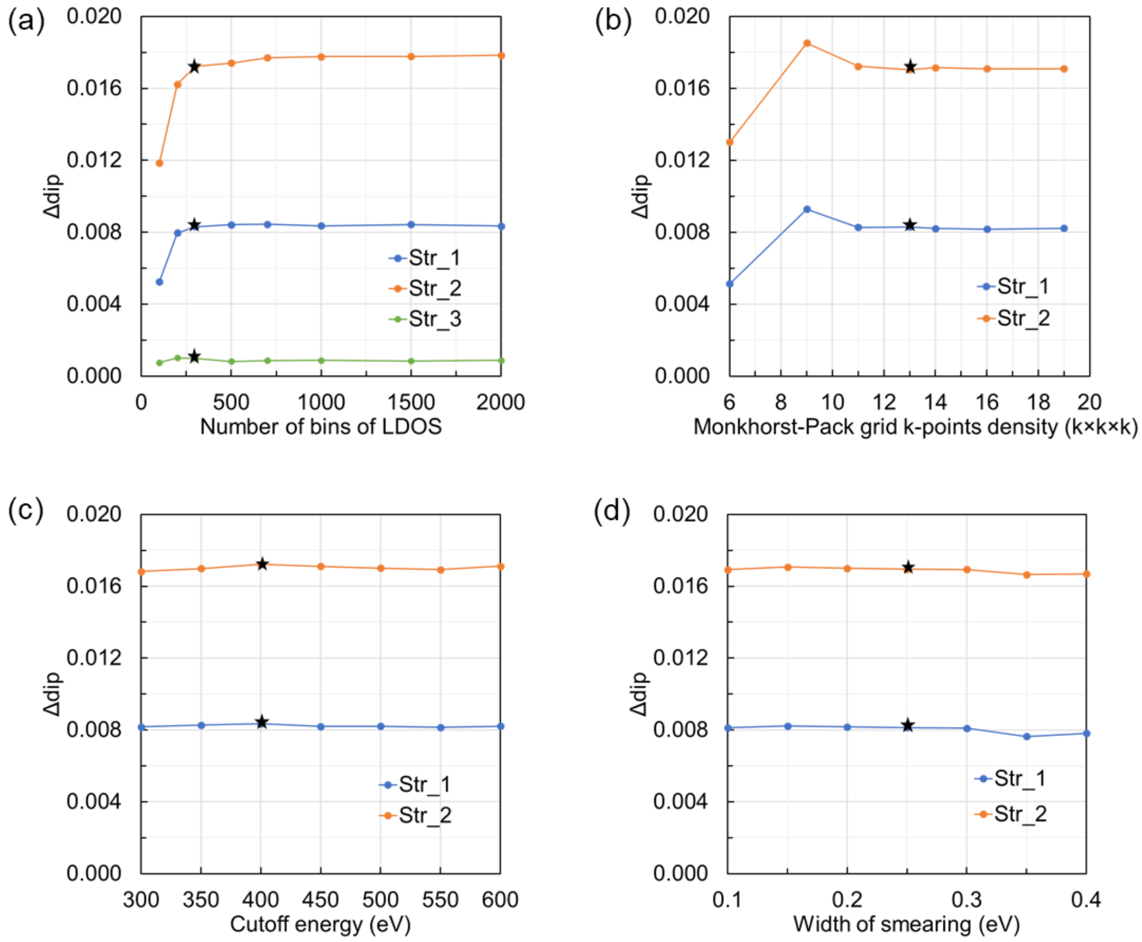

Supplementary Figure 8. Δdip of the perturbated atom (red) in Supplementary Figure 7 with respect to the variations of the values of the LDOS-related DFT parameters. (a) Number of bins of LDOS; (b) the density of the *k*-point mesh; (c) the cutoff energy for the plane-wave basis; (d) the width of the Methfessel-Paxton smearing. The parameter values used for the defect calculations in the main text are marked by a star symbol in each sub-figure.

The effects of the density of k-point mesh on the Δdip measurement are also investigated. As shown in Supplementary Figure 8, Δdip reaches a converged value when the k-point sampling is denser than 11×11×11. For the defect calculations in the present work, the grid of the k-point mesh is set according to the supercell size to reach an overall k-point density similar to that of the 13×13×13 grid in Supplementary Figure 8. In addition, as shown in Supplementary Figures 8c and 8d, the Δdip measurements are insensitive to the variations in the setting of the cutoff energy for the plane-wave basis and the width of the smearing.

Supplementary Note 2

Results of $E_{\text{int}}^{\text{fix}}$, Δdip and $x_{\text{sp}}$from first-principles calculations

It should be noted that the values of Δdip parameter are calculated from the statistical method mentioned in the Methods section in the main text. The random number generator with varying seeds used in the statistical method may cause tiny statistical uncertainties in the calculated Δdip values. We therefore performed test calculations using different pseudo-random number sequences to calculate Δdip values for all defect sites. Then we tested the linear regression model based on Eq. (1) in the main text for the W-Re and W-Pt systems because they have large regression coefficients of Δdip as shown in Table 1 in the main text. We found the model produces almost identical results for predicting $E_{\text{int}}^{\text{fix}}$. The changes in RMSE of the regression results for both cases are always about 0.001 eV or less. Here, to ensure the Δdip values in Supplementary Tables 2 and 3 are exactly reproducible, we used a fixed pseudo-random number sequence to calculate them from the d-band LDOS. This fixed pseudo-random number sequence can be retrieved from the code uploaded on the open-access repositories mentioned in the sections of Data availability and Code availability. We emphasize that such constrain on the random generator and pseudo-random number sequence is not mandatory but only for the purpose of data reproduction and verification. Regression results with the same accuracy can still be achieved with flexible random number sequences.

Supplementary Table 2. Calculated Δdip and x_{sp} of the crystalline defects in bcc W along and their fixed-lattice interaction energies ($E_{\text{int}}^{\text{fix}}$) with the Ta, Re and Pt solutes. The results are listed for each defect site of interest, which is marked by numbers in Supplementary Figures 1-4.

Defect geometry	Site number	Δdip	x_{sp}	$E_{\text{int}}^{\text{fix}}$ (eV)		
				**Ta**	**Re**	**Pt**
Mono-vacancy	1	0.01832	0.07198	-0.0998	0.2263	0.8757
	2	0.00713	-0.04748	-0.1840	0.1806	0.2236
	3	0.00172	-0.00679	0.0001	0.0149	0.0142
	4	0.00073	-0.00309	-0.0022	0.0212	0.0981

	5	0.00563	-0.00406	-0.0297	0.0310	0.0903
	6	-0.00343	-0.00024	0.0269	-0.0286	-0.0628
<100>-dumbbell	1	0.02997	-0.34620	-0.7279	0.9235	2.7096
	2	0.02362	-0.04405	-0.1812	0.3015	0.8689
	3	0.01317	-0.00895	-0.1023	0.2219	0.9095
	4	-0.00177	0.09142	0.2147	-0.1238	0.2453
	5	0.00073	0.02582	0.0592	-0.0150	0.2429
	6	0.00568	-0.00652	-0.0664	0.0806	0.2829
	7	0.00143	-0.01575	-0.0525	0.0394	0.0743
<111>-dumbbell	1	0.03050	-0.26858	-0.6675	0.7907	2.1882
	2	0.01165	0.02768	-0.0372	0.1561	0.7601
	3	0.00704	0.01912	-0.0067	0.0959	0.5490
	4	0.00975	0.02092	-0.0414	0.1164	0.5651
	5	0.00518	0.01162	-0.0127	0.0731	0.3682
	6	0.00232	0.00240	-0.0509	0.0505	0.1511
	7	0.00175	0.00528	-0.0383	0.0469	0.1490
$\frac{1}{2}\langle 111\rangle$ screw dislocation	1	0.01388	0.00124	-0.0445	0.2098	1.1231
	2	0.00525	-0.00372	-0.0209	0.0874	0.5958
	3	0.00329	0.00167	0.0020	0.0407	0.4744
	4	0.00138	-0.00190	-0.0068	0.0135	0.0447
	5	0.00262	-0.00289	-0.0250	0.0396	0.2104
	6	0.00016	-0.00083	-0.0079	0.0177	0.0770
	7	0.00057	0.00030	0.0039	-0.0041	-0.0034
	8	-0.00007	0.00006	0.0014	-0.0012	-0.0045
$\Sigma3(11\bar{2})$ TB	1	0.00957	0.00651	-0.0265	0.1793	0.5477
	2	0.01304	-0.02002	-0.1218	0.2396	0.7446
	3	0.00317	-0.01764	-0.0823	0.1057	0.1944
	4	0.00071	-0.01923	-0.0734	0.0661	0.0152
	5	-0.00067	-0.01059	-0.0402	0.0295	-0.0596
	6	-0.00025	-0.00409	-0.0138	0.0152	-0.0018
	7	-0.00001	-0.00110	0.0031	0.0045	0.0010
	8	-0.00034	-0.00028	0.0063	-0.0002	0.0122
	9	-0.00013	-0.00022	0.0010	-0.0003	-0.0090

Σ3(111) GB	1	0.02362	0.15635	0.2010	0.1923	1.3580
	2	0.02108	-0.15001	-0.5423	0.6293	1.4171
	3	0.01110	0.06491	-0.0848	0.2361	0.9215
	4	0.00521	-0.01007	-0.0651	0.1181	0.1814
	5	0.00219	-0.03459	-0.1109	0.1169	-0.0279
	6	0.00316	-0.01272	-0.0773	0.0703	0.0738
	7	0.00224	-0.00382	-0.0317	0.0338	-0.0314
	8	0.00130	-0.00209	-0.0503	0.0533	0.0383
Σ5(310) GB	1	0.02262	0.11231	0.1472	0.1079	0.8986
	2	0.03008	-0.01120	-0.2484	0.4839	1.5242
	3	0.01176	-0.01821	-0.1457	0.2291	0.7396
	4	0.00567	-0.02215	-0.1108	0.1100	0.2283
	5	0.00209	-0.01255	-0.0587	0.0697	0.1439
	6	-0.00348	-0.00348	-0.0430	-0.0009	-0.1392
	7	-0.00022	-0.00322	-0.0340	0.0154	-0.0327
	8	0.00082	-0.00026	-0.0201	0.0133	0.0496
	9	-0.00060	-0.00041	-0.0141	-0.0118	-0.0115
	10	-0.00041	-0.00283	-0.0175	-0.0069	-0.0202
Σ5(210) GB	1	0.02845	0.10837	-0.0312	0.3382	1.7014
	2	0.03101	0.02800	-0.2155	0.5387	1.8451
	3	0.03033	-0.16447	-0.5172	0.6986	1.7709
	4	0.01970	-0.00256	-0.2326	0.3729	1.2487
	5	0.01563	0.13434	0.1485	0.0573	1.0862
	6	0.01205	0.00592	-0.0824	0.1928	0.7215
	7	0.01199	-0.01932	-0.1470	0.2319	0.7663
	8	0.00712	-0.03905	-0.1972	0.2176	0.5459
	9	0.00796	-0.01194	-0.0890	0.1353	0.3926
Σ13(230) GB	1	0.02680	0.17293	0.2524	0.1096	1.6310
	2	0.02807	-0.08316	-0.3524	0.6173	1.7313
	3	0.02398	0.03149	-0.1157	0.3907	1.8286
	4	0.01725	0.12157	0.0859	0.1860	1.5786
	5	0.01339	0.18143	0.1711	0.0856	1.3288
	6	0.01913	-0.00892	-0.1016	0.2428	0.9261

	7	0.00959	-0.05661	-0.1790	0.2133	0.4672
	8	0.01200	-0.01811	-0.0674	0.1557	0.6563
	9	0.00515	0.00518	-0.0175	0.0698	0.4478
	10	0.00918	0.01765	-0.0018	0.0621	0.3913
	11	0.00304	0.00711	0.0139	0.0408	0.2973
Σ27(552) GB	1	0.03368	-0.06269	-0.4185	0.7044	2.3040
	2	0.02173	0.16544	0.1239	0.2366	1.4783
	3	0.02974	-0.10847	-0.4381	0.5984	1.5620
	4	0.02566	-0.07426	-0.3027	0.4664	1.2015
	5	0.00121	0.12667	0.2516	-0.0721	0.5076
	6	0.02538	-0.04973	-0.2276	0.4457	1.3732
	7	0.03203	-0.09782	-0.4614	0.6646	1.8454
	8	0.00138	0.12821	0.1775	0.0081	0.7234
	9	0.02547	-0.13807	-0.3932	0.5925	1.5470
	10	-0.00201	-0.03070	-0.1488	0.0580	0.0621
	11	0.00917	0.10158	0.2249	-0.0039	0.6385
	12	0.01671	0.03534	-0.0167	0.2170	0.9612
	13	0.01113	-0.09382	-0.3179	0.3884	0.7847
	14	0.01156	-0.00181	-0.1278	0.2163	0.5038
	15	0.01160	-0.00164	-0.1277	0.2164	0.5047

Supplementary Table 3. Calculated Δdip and x_{sp} of the crystalline defects in bcc Ta along with their fixed-lattice interaction energies with the Hf and Os solutes. The results are listed for each defect site of interest, which is marked by numbers in Supplementary Figures 1-4.

**Defect geometry**	**Site number**	**Δdip**	x_{sp}	$E_{\mathrm{int}}^{\mathrm{fix}}$ **(eV)**	
				Hf	Os
Mono-vacancy	1	0.02507	0.13836	0.3505	-0.5476
	2	0.00257	-0.02318	-0.0800	0.2739
	3	0.00408	0.00949	0.0362	-0.0860
	4	-0.00431	-0.00215	-0.0503	0.0627
	5	0.00450	0.01024	0.0348	-0.0658
	6	0.00342	0.00683	0.0596	-0.0663

<100>-dumbbell	1	0.03041	-0.16101	0.0595	0.2425
	2	0.01848	0.02479	0.0952	-0.1558
	3	0.01130	0.02542	0.1342	-0.2050
	4	0.00078	0.10643	0.3034	-0.6644
	5	-0.00007	0.03927	0.1083	-0.2639
	6	0.00448	0.00455	0.0311	-0.0914
	7	0.00030	-0.00784	-0.0216	-0.0230
<111>-dumbbell	1	0.02274	-0.13813	-0.1085	0.3606
	2	0.01143	0.05669	0.2239	-0.3557
	3	0.00715	0.04369	0.1614	-0.2729
	4	0.00903	0.05746	0.2143	-0.2868
	5	0.00270	0.02742	0.0965	-0.1774
	6	0.00229	0.00172	0.0271	-0.0615
	7	0.00090	0.00226	0.0213	-0.0426
$\frac{1}{2}\langle 111\rangle$ screw dislocation	1	0.00987	0.05393	0.2065	-0.3910
	2	0.00170	0.02305	0.0853	-0.1225
	3	0.00313	0.02401	0.0749	-0.1278
	4	0.00117	0.00132	-0.0074	0.0506
	5	0.00080	0.01132	0.0487	-0.0273
	6	-0.00077	0.00271	0.0115	0.0492
	7	0.00043	0.00015	-0.0024	0.0753
	8	0.00046	-0.00137	-0.0064	0.0732
$\Sigma 3(11\bar{2})$ TB	1	0.01171	0.04554	0.1527	-0.2527
	2	0.01341	0.02931	0.1222	-0.2363
	3	-0.00302	0.00853	0.0016	-0.0744
	4	0.00034	0.00415	0.0036	-0.0740
	5	-0.00139	0.00354	0.0011	-0.0160
	6	-0.00136	0.00326	0.0073	-0.0043
	7	-0.00096	0.00355	0.0076	0.0021
	8	-0.00092	0.00132	-0.0035	0.0093
	9	-0.00063	0.00026	-0.0044	0.0063
$\Sigma 3(111)$ GB	1	0.02300	0.19686	0.6176	-1.0476
	2	0.01853	-0.05261	-0.0330	0.1966

	3	0.00668	0.15796	0.4496	-0.6445
	4	0.00343	0.04247	0.1174	-0.4448
	5	-0.00092	-0.00605	-0.0471	-0.1087
	6	0.00204	0.01151	0.0300	-0.1471
	7	0.00034	-0.00079	0.0203	-0.0861
	8	-0.00178	-0.00731	-0.0251	0.0076
Σ5(310) GB	1	0.01089	0.16921	0.4894	-0.7209
	2	0.02846	0.07849	0.2651	-0.2182
	3	0.00434	0.05210	0.0860	-0.2069
	4	0.00220	-0.01014	-0.0234	-0.2595
	5	0.00306	0.02196	0.1088	-0.2534
	6	-0.00084	-0.00024	-0.0018	-0.0706
	7	0.00057	-0.00153	-0.0002	-0.0117
	8	-0.00057	-0.00277	-0.0188	-0.0119
	9	0.00093	-0.00125	0.0033	-0.0298
	10	0.00049	0.00023	0.0044	-0.0186
Σ5(210) GB	1	0.02145	0.17245	0.4808	-0.7458
	2	0.02661	0.07989	0.2989	-0.4672
	3	0.02599	-0.08966	-0.0220	0.2253
	4	0.01732	0.15659	0.4545	-0.7319
	5	0.00392	0.18039	0.4542	-0.8383
	6	0.01918	0.01762	0.1310	-0.3117
	7	0.01209	0.11985	0.3565	-0.7699
	8	-0.00088	-0.01514	-0.0986	-0.1471
	9	0.00549	0.03848	0.1445	-0.4000
Σ13(230) GB	1	0.02617	0.24272	0.7799	-1.4030
	2	0.02768	0.04111	0.2764	-0.1480
	3	0.02237	0.13355	0.4409	-0.6619
	4	0.01813	0.17526	0.5431	-0.9401
	5	0.02110	0.23624	0.7173	-1.3073
	6	0.02112	0.05575	0.3220	-0.5296
	7	0.00911	-0.02219	-0.0944	-0.0974
	8	0.01058	0.01825	0.0944	-0.4580

	9	0.00438	0.03169	0.1264	-0.4528
	10	0.01623	0.06708	0.2847	-0.6127
	11	0.00487	0.02080	0.0835	-0.2411
	1	0.03065	0.09628	0.2970	-0.1672
	2	0.01727	0.26328	0.7191	-1.2106
	3	0.02050	0.02095	0.0838	-0.1147
	4	0.02135	0.02014	0.1649	-0.3441
	5	-0.00098	0.20705	0.5807	-1.2218
	6	0.02268	0.05130	0.2049	-0.4531
Σ27(552)	7	0.02475	0.04125	0.1649	-0.1006
GB	8	-0.00113	0.20682	0.5094	-0.9953
	9	0.02184	-0.03558	0.0205	0.0270
	10	-0.00763	0.00894	-0.0189	-0.1196
	11	0.00722	0.15381	0.4511	-1.0681
	12	0.01039	0.10476	0.3202	-0.6625
	13	0.00511	0.00228	0.0043	-0.0530
	14	0.00599	0.04009	0.1433	-0.4048
	15	0.00606	0.04025	0.1439	-0.4057

Supplementary Note 3

Comparison between $\boldsymbol{E}_{\mathbf{int}}^{\mathbf{relax}}$ and $\boldsymbol{E}_{\mathbf{int}}^{\mathbf{fix}}$

To evaluate the effects of the lattice relaxation on the solute-defect interaction, the "*relaxed interaction energies*" ($E_{\text{int}}^{\text{relax}}$), which were obtained by full relaxation of atomic positions due to solute substitutions, are also calculated for a few of defect sites that have relatively strong interactions with the solutes. The difference between the relaxed ($E_{\text{int}}^{\text{relax}}$) and fixed-lattice interaction energies ($E_{\text{int}}^{\text{fix}}$) gives the energy change due to the relaxation of the defect lattice upon the solute substitution. As shown in Fig. 1a in the main text, in W alloys, the relative difference between $E_{\text{int}}^{\text{relax}}$ and $E_{\text{int}}^{\text{fix}}$ of the solute-dislocation interactions is small, which indicates that the interactions may mainly originate from the changes in the local electronic bonding environment near the defects compared to the bulk lattice. It is worth to further test whether the difference between $E_{\text{int}}^{\text{relax}}$ and $E_{\text{int}}^{\text{fix}}$ is also small or not for other types of solute-defect interactions in the Ta and W alloys. Here, the Ta-Hf and W-Pt systems are chosen as two samples, in which the relaxed interaction energy ($E_{\text{int}}^{\text{relax}}$) is calculated for a few defect sites that have relatively strong interactions with the solutes. The calculated results are summarized in Supplementary Tables 4 and 5 for the Ta-Hf and W-Pt systems, respectively. The corresponding values of $E_{\text{int}}^{\text{fix}}$ are also included in the tables for comparison. As shown in Supplementary Tables 4 and 5, the difference between $E_{\text{int}}^{\text{relax}}$ and $E_{\text{int}}^{\text{fix}}$ is generally small for various types of solute-defect interactions in the Ta-Hf and W-Pt alloys. Along with the results of Fig. 1a, it suggests that the relaxation of the atomic positions generally has minor effects on the solute-defect interactions in the bcc refractory alloys for substitution sites close (<= ~1 nm) to defect centers. The interactions mainly originate from the variations in the local electronic structures due to the presence of the defect geometry.

Supplementary Table 4. Calculated $E_{\mathrm{int}}^{\mathrm{relax}}$ and $E_{\mathrm{int}}^{\mathrm{fix}}$ of the interactions between Hf and the crystalline defects in bcc Ta. (unit: eV)

	**Vacancy**	**<111>-dumbbell**	**Σ3 $(11\bar{2})$ twin boundary**	**Σ5 (310) grain boundary**
Site*	1-nn	2-nn	1-nn	1-nn
$E_{\mathrm{int}}^{\mathrm{relax}}$	0.388	0.213	0.138	0.499
$E_{\mathrm{int}}^{\mathrm{fix}}$	0.351	0.223	0.152	0.489

*1-nn: the atomic site with the first shortest distance to the defect center; 2-nn: the atomic site with the second shortest distance to the defect center

Supplementary Table 5. Calculated $E_{\mathrm{int}}^{\mathrm{relax}}$ and $E_{\mathrm{int}}^{\mathrm{fix}}$ of the interactions between Pt and the crystalline defects in bcc W. (unit: eV)

	**Vacancy**		**Σ3 $(11\bar{2})$ twin boundary**		**Σ5 (310) grain boundary**	
Site*	1-nn	2-nn	1-nn	2-nn	1-nn	2-nn
$E_{\mathrm{int}}^{\mathrm{relax}}$	0.947	0.184	0.754	0.921	0.851	1.675
$E_{\mathrm{int}}^{\mathrm{fix}}$	0.876	0.224	0.548	0.745	0.899	1.524

*1-nn: the atomic site with the first shortest distance to the defect center; 2-nn: the atomic site with the second shortest distance to the defect center.

Supplementary Note 4

Correlation between the bimodality of LDOS and the solute-GSF interactions in bcc W

The variations in the electronic structure of the atom on the fault plane of the GSFs in pure bcc W are also investigated via first-principles calculations. Δdip and x_{sp} of the W atom on the fault plane are calculated as a function of the relative displacement of the $[111](1\bar{1}0)$ and $[111](11\bar{2})$ GSF and summarized in Supplementary Table 6 below.

Supplementary Table 6. Calculated Δdip and x_{sp} of the W atom on the fault plane of the $[111](1\bar{1}0)$ and $[111](11\bar{2})$ GSF with different slip displacements, along with the fixed-lattice interaction energies when the W atom is substituted by the Re or Pt solutes. The value of the relative displacement along the slip direction is relative to the length of Burgers vector, which is 1/2[111].

GSF geometry	Relative displacement	Δdip	x_{sp}	$E_{\mathrm{int}}^{\mathrm{fix}}$ (eV)	
				Re	Pt
$[111](1\bar{1}0)$	0.1	0.00303	0.00916	0.0040	0.1590
	0.2	0.00872	0.01580	0.0609	0.4899
	0.3	0.01314	0.01694	0.1264	0.7502
	0.4	0.01500	0.01439	0.1827	0.9053
	0.5	0.01506	0.01545	0.2037	0.9556
	0.6	0.01500	0.01439	0.1827	0.9053
	0.7	0.01314	0.01694	0.1264	0.7502
	0.8	0.00872	0.01580	0.0609	0.4899
	0.9	0.00303	0.00916	0.0040	0.1590
$[111](11\bar{2})$	0.1	0.00365	0.01020	0.0130	0.2223
	0.2	0.01040	0.01124	0.1195	0.8254
	0.3	0.01718	-0.00545	0.2766	1.2738
	0.4	0.01734	-0.00078	0.3158	1.3629
	0.5	0.01495	0.01327	0.2707	1.2009
	0.6	0.01496	0.01860	0.2220	1.0360
	0.7	0.01564	0.01934	0.2189	1.0368
	0.8	0.01364	0.01865	0.1723	0.9180
	0.9	0.00672	0.01554	0.0519	0.4331

As shown in Supplementary Table 6, the creation of GSF results in variations in the local electronic structure of the atom on the fault plane, associated with an increase of the Δdip parameter. In addition, $E_{\mathrm{int}}^{\mathrm{fix}}$ are also calculated for the Re and Pt solutes. Supplementary Figures 9a and 9b plotted the calculated Δdip and $E_{\mathrm{int}}^{\mathrm{fix}}$ with respect to

the relative displacement along the slip direction for the $[111](1\bar{1}0)$ and $[111](11\bar{2})$ GSF, respectively. As shown in Supplementary Figures 9a and 9b, the shape of the interaction-energy curves is very similar to that of the Δdip curves, for both Re and Pt, indicating the strong correlation between the LDOS bimodality and the solute-defect interactions. Furthermore, it is found that this correlation can be extended to the defect geometries that are artificially frozen in unstable states. Supplementary Figure 10a shows a contour plot of the Δdip for the GSF of the entire $(1\bar{1}0)$ plane in pure W. Correspondingly, as shown in Supplementary Figure 10b, the contour plot of $E_{\mathrm{int}}^{\mathrm{fix}}$ of Re exhibits a similar gradient change as that of Δdip.

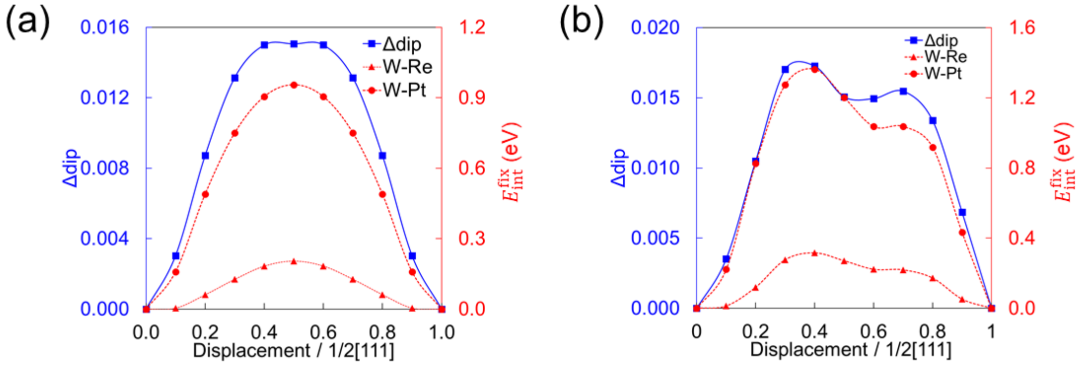

Supplementary Figure 9. Changes in bimodalities of LDOSs (Δdip) of the atom on fault plane with respect to the relative displacement of the GSFs in pure W, along with the corresponding solute-defect interaction energies ($E_{\mathrm{int}}^{\mathrm{fix}}$) when the W atom is substituted by a solute atom (Re and Pt). (a) $[111](1\bar{1}0)$ GSF; (b) $[111](11\bar{2})$ GSF.

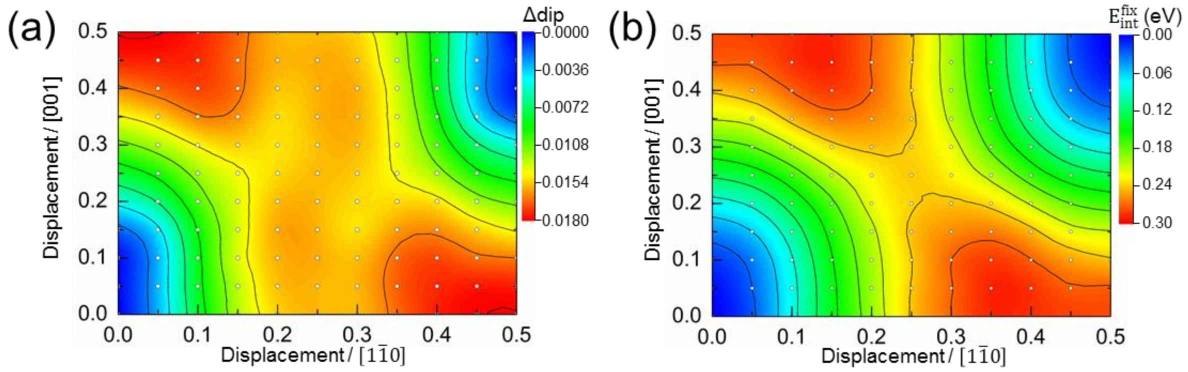

Supplementary Figure 10. (a) Contour plot of Δdip of the atom on the defect plane of the GSF of the entire $(1\bar{1}0)$ plane in pure W. (b) Contour plot of $E_{\mathrm{int}}^{\mathrm{fix}}$ when one W atom on the defect plane is substituted by a Re atom. The white dots in both figures represent the slip displacements where the first-principles calculations are performed. The coloring between each white dot is a linear-interpolation of the DFT results.

Supplementary Figure 11

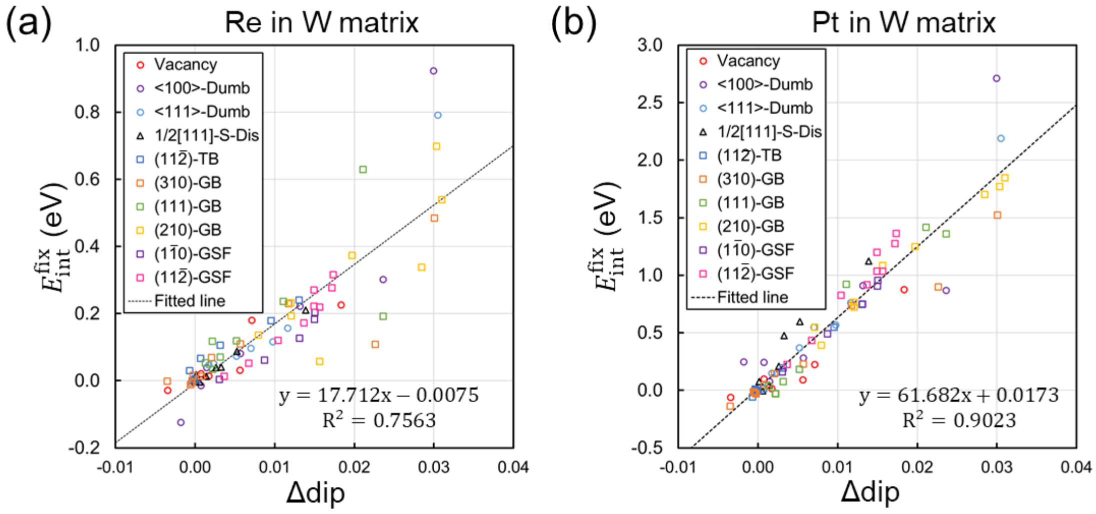

Supplementary Figure 11. General correlation between Δdip and $E_{\mathrm{int}}^{\mathrm{fix}}$ in the W-Re and W-Pt binary alloys. Linear correlations between $E_{\mathrm{int}}^{\mathrm{fix}}$ and Δdip in the W-Re and W-Pt binary alloys. The calculated solute-defect interaction energies ($E_{\mathrm{int}}^{\mathrm{fix}}$) in W are plotted with respect to the corresponding Δdip parameters for different atomic sites near various types of defects. (a) Re as the solute; (b) Pt as the solute.

Supplementary Note 5

Hybridization between the valence *sp*- and *d*-bands in bcc W

Beside the importance of the *d*-band, the energy contributions from the valence *sp*-band were found to be crucial in accurately characterizing many fundamental physical properties of transition metal elements, such as cohesive energy[9], equilibrium atomic volume[10,11] and bulk modulus[10,11]. On the one hand, the valence *s*-band strongly overlaps and hybridizes with the valence *p*-band, even though the *p*-band is initially unoccupied in the free-atomic state[12]. On the other hand, both the *s*- and *p*-bands are strongly influenced by the presence of the *d*-band. For example, the valence *sp* electrons may be squeezed into the ion core region due to the large covalent *d*-bonding forces, which results in necessary repulsive forces against the strongly attractive *d*-contributions to maintain the equilibrium atomic distance[10,12]. In addition, it was pointed out that the hybridization between the valence *d*-band and *sp*-band results in large changes in the electronic density of states (DOS), which could provide a significant effect on the cohesive energy in the noble and transition metals[9]. Supplementary Figure 12 shows the LDOS of the *s*-, *p*- and *d*-bands of perfect bcc W. As seen from the figure, the peaks of *s*- and *p*-bands are largely overlapped with that of the *d*-bands, which qualitatively indicates a hybridization behavior between the valence *sp*-band and *d*-band.

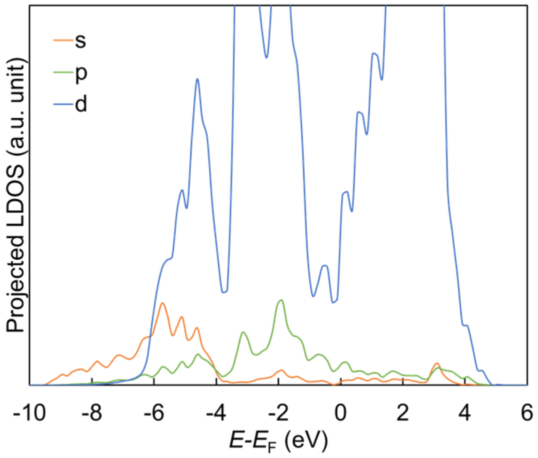

Supplementary Figure 12. Projected LDOS of the *s*-, *p*- and *d*-band of W atoms in perfect bcc lattice with equilibrium lattice parameter. It should be noted that the pseudopotential used for the calculation also treats the semi-core *5p* electrons as valence electrons. However, the band from *5p* electrons is localized at very low energy far away from the Fermi level, thus it is not plotted here.

Based on the bond-order potential theory, the influence of the *d*-band on *sp*-band can be screened in terms of the local density and environment that can be expressed as a function of interatomic distances[13,14]. To view the variations in the valence *sp*-band with respect to interatomic distances, Supplementary Figure 13 shows the LDOS of the valence *6s* and *6p* bands of W atoms in the perfect bcc lattice with different lattice parameters. In addition, the LDOS of a W atom in unbonded state, which can be considered as the infinite large interatomic distance, is also included for comparison. As seen from Supplementary Figures 13a and 13b, before bonding, the LDOSs of both *s*- and *p*-band are single peaks since no hybridization occurs (brown solid-line). Also, the *6p*-band is unoccupied as it is above the Fermi level. As W atoms are bonded in a perfect bcc lattice, their *6s*- and *6p*-bands become broad due to the effect of hybridization. In addition, because of the charge transfer caused by hybridization, the initially unoccupied *6p*-band becomes partially occupied. Furthermore, as shown in Supplementary Figure 13, the *sp*-band becomes broader when the interatomic distance is shorter (i.e. smaller lattice parameter), implying a stronger hybridization effect. This can be anticipated because a shorter interatomic distance results in larger *d*-bond integrals, thus stronger covalent *d*-bonding forces[13,14].

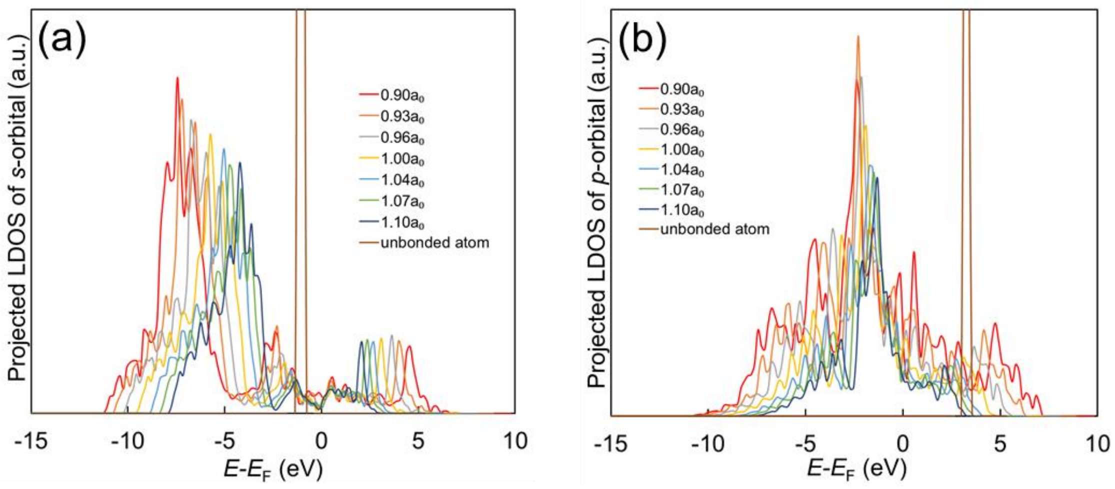

Supplementary Figure 13. The LDOS of of W atoms in the perfect bcc lattice but with different lattice parameters. (a) *6s* band; (b) *6p* band. The length of the equilibrium lattice parameter of bcc W is represented as a_0 in the legend.

According to an energy band model developed by Hodges[9], Mueller[15] and Pettifor[10,16], the strength of the hybridization between the valence *sp* and *d* bands in transition metal elements is propositional to the width of the local *d*-band. Furthermore, the local *d*-band

width of an atom is closely related to the magnitude of the *d*-*d* interaction matrix elements (V_{dd}^{ij}) between the atom and its neighboring atoms[17,18]. The relationship is written as[17,18],

$$W_i \propto \sum_j V_{\mathrm{dd}}^{ij} \tag{1}$$

where *j* represents the neighboring atoms of atom *i*, W_i is the local *d*-band width and V_{dd}^{ij} is the *d*-*d* interaction matrix element between atom *i* and *j*, which can be scaled as[18,19],

$$V_{\mathrm{dd}}^{ij} \propto \frac{r_{d_i}^{\frac{3}{2}} r_{d_j}^{\frac{3}{2}}}{d_{ij}^5} \tag{2}$$

where d_{ij} is the interatomic distance between atom *i* and *j*, and r_{d_i} is the spatial extent of *d*-orbital of atom *i*, which is an intrinsic element-property[18]. Therefore, the strength of the *sp*-*d* hybridization (E_{sp}) of an atom *i* in transition metal alloys can be estimated as,

$$E_{\mathrm{sp}} \propto W_i \propto \sum_j V_{dd}^{ij} \propto \sum_j r_{d_i}^{\frac{3}{2}} r_{d_j}^{\frac{3}{2}} / d_{ij}^5. \tag{3}$$

The above derivation suggests that the strength of the *sp*-*d* hybridization in a defect structure is expected to vary for each individual atom, because the interatomic distances (d_{ij}) of each defect site can be different due to the presence of the defect geometry and the spatial extent of *d*-orbitals of the solute element (r_{d_i}) can be different from that of the matrix element. Therefore, the effects of the *sp*-*d* hybridization may not be ignored to understand the solute-defect interactions in the bcc refractory alloys.

Supplementary Note 6

Supplementary details of the construction of the x_{sp} parameter

In the present work, x_{sp} of an atom near the defect in pure metals is proposed to be,

$$x_{\mathrm{sp}} = 1 - \frac{\left(V_{\mathrm{vor}}^{\mathrm{def}}\right)^{-\frac{5}{3}} / \epsilon_{\mathrm{sp}}^{\mathrm{def}}}{\left(V_{\mathrm{vor}}^{\mathrm{ref}}\right)^{-\frac{5}{3}} / \epsilon_{\mathrm{sp}}^{\mathrm{ref}}} \quad (4)$$

where $V_{\mathrm{vor}}^{\mathrm{def}}/V_{\mathrm{vor}}^{\mathrm{ref}}$ is the Voronoi volume of the atom at the defect and reference site, respectively, and $\epsilon_{\mathrm{sp}}^{\mathrm{def}}$ /$\epsilon_{\mathrm{sp}}^{\mathrm{ref}}$is the center of the occupied *sp*-band projected on the atom at the defect and the reference site, respectively. The reference site is same as the one used for the calculation of $\Delta\mathrm{dip}$ and $E_{\mathrm{int}}^{\mathrm{fix}}$. The $\epsilon_{\mathrm{sp}}^{\mathrm{def}}$ term is calculated as,

$$\epsilon_{\mathrm{sp}}^{\mathrm{def}} = \int_{-\infty}^{0} E\rho_{\mathrm{sp}}^{\mathrm{def}}(E)dE \,/ \int_{-\infty}^{0} \rho_{\mathrm{sp}}^{\mathrm{def}}(E)dE \quad (5)$$

where $\rho_{\mathrm{sp}}^{\mathrm{def}}(E)$ is the projected LDOS of the *sp*-band on the atom at the defect site and the Fermi energy E_F is set to zero. $\epsilon_{\mathrm{sp}}^{\mathrm{ref}}$ is calculated in the same way for the atom at the reference site. The Voronoi volume and LDOS of the *sp*-band are calculated from the relaxed atomic structures of pure matrix metals that contain defects. In Supplementary Equation 4, Voronoi volume (V_{vor}) is used to describe the average changes in the interatomic distances (d_{ij}) of the atoms near the defect. The exponent value, $-\frac{5}{3}$, is obtained since E_{sp} is proportional to $\left(d_{ij}\right)^{-5}$, as shown by Supplementary Equation 3. A benefit of using Voronoi volume instead of directly calculating d_{ij} is to avoid arbitrary assignment of the cutoff distance for identifying neighboring atoms. In Supplementary Equation 4, we also include a scaling term, $1/\epsilon_{\mathrm{sp}}$. Based on the definition (Supplementary Equation 5), ϵ_{sp} actually characterizes the average difference between the energy states of the *sp*-band and E_F. Generally speaking, the electrons with energies close to E_F may be more sensitive to small perturbations due to atomistic structure variations. We thus propose that, for the *sp*-band closer to the E_F, its corresponding *sp*-*d* hybridization may have a stronger effect on the ground state energy. Therefore, the inverse of ϵ_{sp} is used to scale the effects of *sp*-*d* hybridization on solute-defect interactions. It is noteworthy that the inclusion of the ϵ_{sp} term in Supplementary Equation 4 is necessary, because we found that the Voronoi volume alone is insufficient

to construct the x_{sp} term that yields an accurate description of the solute-defect interaction energy.

Supplementary Note 7

Performance of the two-descriptor linear model on predicting the $E_{\text{int}}^{\text{fix}}$ calculated from the four-supercell method

Taking the W-Re system as an example, a corresponding investigation was performed for the interaction energies that were calculated using the four-supercell method[20,21]. A 3×3×3 bulk bcc supercell is used as the reference configuration. The solute-defect interaction is then calculated as, $E_{\text{int}}^{\text{fix}} = \left(E_{\text{def}}^{\text{W}} + E_{\text{ref}}^{\text{X}}\right) - \left(E_{\text{def}}^{\text{X}} + E_{\text{ref}}^{\text{W}}\right)$, where $E_{\text{def}}^{\text{X}}$ (respectively $E_{\text{def}}^{\text{W}}$) is the total energy of the defect supercell containing (respectively not containing) the solute atom and $E_{\text{ref}}^{\text{X}}$ (respectively $E_{\text{ref}}^{\text{W}}$) is the energy of the bulk bcc supercell with (respectively without) the solute atom. Correspondingly, Δdip and x_{sp} of the defect atoms are also recalibrated using a W atom in the bulk bcc supercell as the reference, instead of using a W atom in the defect-containing supercell as the reference in the two-supercell method.

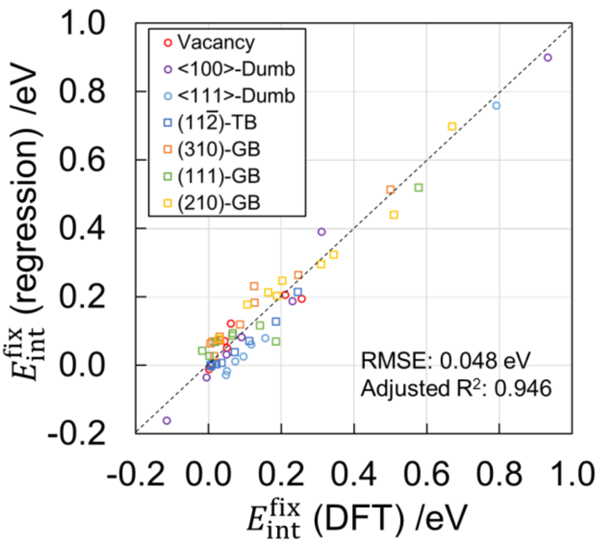

Supplementary Figure 14. Comparison between the $E_{\text{int}}^{\text{fix}}$ calculated from the four-supercell method and predicted from the regression model. The calculations are performed for the W-Re system.

The regression result is shown by a parity plot in Supplementary Figure 14 above. It is important to note that the four-supercell calculation is not performed for the ½<111> screw dislocation due to the limitation of the flexible boundary condition method specifically used for dislocation core calculations. As shown in Supplementary Figure

14, the interaction energies calculated by the four-supercell method can still be well described by the linear correlation proposed in the present work. The regression coefficients of the Δdip and x_{sp} parameters are 14.21 eV and -1.42 eV, respectively, which are slightly different from the values obtained by the two-supercell method (15.97 eV and -1.29 eV, respectively). This is because different reference states are used to calculate the interaction energies and electronic descriptors in the two methods. On the one hand, using the two-supercell method, both of the defect and reference atoms are in a same supercell containing the defect structure. As a result, the defect-induced and supercell-size-induced strain effects are largely excluded in the calculations of the interaction energy and the $\Delta \mathrm{dip}$ and x_{sp} parameters. On the other hand, the contribution of these strain effects is included in the four-supercell calculations as the reference atom is now in a perfect bcc lattice supercell with relatively large supercell size. Consequently, the regression coefficients of the four-supercell calculations are slightly different from those obtained by two-supercell method.

Supplementary Note 8

Prediction outliers from the two-descriptors linear model

As discussed in the main text, a few of outliers still appear in the predictions of the two-descriptor linear model (Equation. (1) in main text), which have apparent discrepancies from the DFT results. Interestingly, we found that these outliers usually are at particular defect sites and repeatedly appear in multiple alloying systems. For example, the linear model yields relatively large deviations when predicting the interaction energies of the site 3 of the (111)-GB (Supplementary Figure 3b) in both the W-Ta (an overestimate of 0.120 eV) and W-Re systems (an underestimate of 0.144 eV). Additionally, the solute-interactions of the <100>-dumbbell defect seem to be poorly described in the W-Pt, Ta-Hf and Ta-Os systems, especially the first- and second-nearest sites to the defect center. Further scrutinizing the electronic structures of the outlier defect atoms, it is found that the variations in their LDOSs relative to the reference atom may not be sufficiently characterized by the Δdip and x_{sp} parameters, which results in large errors in predictions of the interaction energy. Details are described in the two following paragraphs.

In Supplementary Figure 15a the *d*-orbital LDOS for a W atom that occupying the site 3 of the (111)-GB (refer as the outlier defect atom) is plotted to compare with the LDOSs of the reference W atom far away from the defect and a W atom at a site near the (210)-GB (referred as the normal defect atom, where the present model yields an accurate prediction on the interaction energy). As illustrated by the shapes of the whole LDOSs curves, both the outlier and normal defect atoms have less bimodal LDOSs compared to the reference atom. Especially, their LDOSs are very close to each other in the region around the bcc pseudo-bandgap. This implies that the contributions of the *d*-band filling effect to the interaction energy should be quite similar for these two atoms when they are substituted by the Ta or Re solutes. Therefore, one would expect the outlier and normal atoms to have a similar value of the Δdip parameter to correctly reflect the *d*-band filling effect. However, in practice, due to the presence of an abnormally higher peak in right side band of the outlier atom, its Δdip is calculated to be 0.0111, much smaller than that of the normal atom, 0.0197. Correspondingly, the

interaction energy of the outlier site is overestimated by the linear model for Ta-substitution and underestimated for Re-substitution. This inconsistency suggests that more descriptors of LDOSs should be included to fully describe their effects. For example, more information can be obtained if the LDOSs of the outlier and normal defect atoms are plotted in the t_{2g} and e_g orbital sets individually. As shown in Supplementary Figure 15b, the abnormally high peak of the outlier atom is mainly originated from the e_g orbital set. Additionally, it can be seen that the LDOS of the t_{2g} set of the two atoms are quite close to each other, which yields a similar value of Δdip if we only perform the measurements on the t_{2g} set. These results suggest that the bimodality variations of the individual t_{2g} and e_g orbital sets may serve as additional descriptors to further reduce the prediction errors for the outlier site.

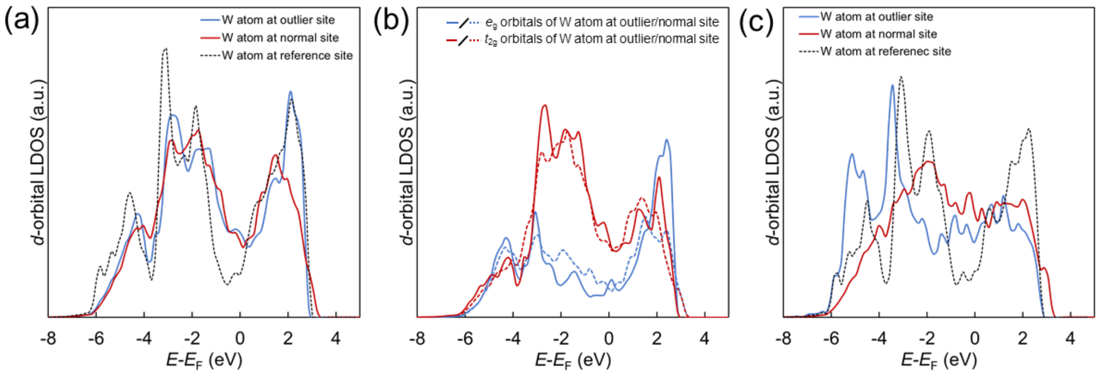

Supplementary Figure 15. (a) the *d*-orbital LDOS for a W atom that occupies the *outlier defect site* of the (111)-GB along with the LDOSs of the reference W atom and a W atom at a *normal defect site* of the (210)-GB, where the present model yields a normal prediction on the interaction energy. (b) The LDOSs of the t_{2g} and e_g orbital sets of the outlier and normal W atoms in (a). (c) the *d*-orbital LDOS for a W atom that occupies the *outlier defect site* of the <100>-dumbbell along with the LDOSs of the reference W atom and a W atom at a *normal defect site* where the present model yields a normal prediction on the interaction energy.

For another set of outliers, the first- and second nearest sites of the <100>-dumbbell defect, we also found that there may be additional features of their LDOSs that are not adequately described by the Δdip and x_{sp} parameters. Similar to Supplementary Figure 15a, in Supplementary Figure 15c we plotted the *d*-orbital LDOS for a W atom that occupies the first-nearest site of the <100>-dumbbell defect along with the LDOSs of the reference W atom and a W atom at a normal defect site, where the linear model yields an accurate prediction on the interaction energy. In addition, it is worth to

mention that the outlier and normal defect atoms have similar values of the Δdip parameters (~0.0300). On the one hand, the LDOS of the outlier atom has a less bimodal shape relative to the LDOS of the reference atom, which is indeed reflected by a positive value of Δdip. On the other hand, the LDOS shapes of the outlier and normal defect atoms are quite different from each other although their Δdip have similar values. As shown in Supplementary Figure 15c, close to the left band edge, there are two sharp peaks in the LDOS of the outlier defect atom, which are not seen in the LDOS of the normal defect atom. Consequently, the effects of these peaks on the solute-defect interaction energy may not be adequately captured by the Δdip measurement, correspondingly resulting in a large prediction error. In addition, another possible reason for the <100>-dumbbell outliers could be the approximation on using the Voronoi volume to represent the average interatomic distance for constructing the x_{sp} parameter. This average may have large uncertainties in terms of the standard deviation when defect-induced lattice distortions become large and anisotropic like the <100>-dumbbell defect. The above results suggest that the remaining residuals of the linear regression model can be reduced if the model includes some other descriptors of the electronic bands in addition to *d*-band bimodality and *sp*-*d* hybridization.

Supplementary Note 9

Algorithm and regression procedures of the residual-correction function $\boldsymbol{f}_{\mathrm{r-c}}(\boldsymbol{D}_i, \boldsymbol{D}_j, \ldots)$

a. Construction of the potential descriptors $\{D_i, D_j, \ldots\}$

As discussed in the main text, the remaining residuals of the linear regression model may result from the energy contributions that correlate with some other local features of the electronic band in addition to the d-band bimodality and *sp*-d hybridization. Therefore, as a first approximation, we considered a broad set of electronic band features as the potential descriptors to construct the residual-correction function, $f_{\mathrm{r-c}}(D_i, D_j, \ldots)$. Similar to $\Delta\mathrm{dip}$ and x_{sp}, all these descriptors $(D_i, D_j, \ldots)$ are also obtained from the DFT calculations of the defects in pure metals for each individual site.

These electronic descriptors are mainly four categories: (i) band center and (ii) root-mean-square width of the LDOS of individual valence orbital sets, including the entire t_{2g} d-orbital set, entire e_g d-orbital set, entire d-orbitals (e_g+t_{2g}), entire s-orbital, entire p-orbitals, entire *sp*-orbitals (LDOSs of s- and p-orbitals are added together), occupied s-orbital, occupied p-orbitals and occupied *sp*-orbitals; (iii) bimodalities of the t_{2g} and e_g d-orbital sets and (iv)Voronoi volume of each site. The band center (ϵ_i) of each orbital set of interest is calculated as,

$$\epsilon_i = \int_{-\infty}^{+\infty} E\rho_i(E)dE \,/\, \int_{-\infty}^{+\infty} \rho_i(E)dE \tag{6}$$

where $\rho_i(E)$ is the projected LDOS of the orbital set i. The root-mean-square width (w_i) is calculated from the second moment of the LDOS, which is,

$$w_i = \sqrt[2]{\int_{-\infty}^{+\infty} (E-\epsilon_i)^2 \rho_i(E)dE \,/\, \int_{-\infty}^{+\infty} \rho_i(E)dE} \tag{7}$$

where ϵ_i is the band center and $\rho_i(E)$ is the projected LDOS of the orbital set i. In addition, in the case of calculations of the occupied states of s-, p- and *sp*-orbitals, the upper bond of the integration of Supplementary Equations 6 and 7 is fixed to be zero, as the Fermi energy E_F is set to zero. The bimodalities of the t_{2g} and e_g d-orbital sets are measured by performing the Hartigan's dip test on their individual LDOS. Furthermore,

similar to the calculation of the Δdip parameter, the value of the descriptor, D_i, of each defect site is taken as, $D_i = x_i^{\mathrm{ref}} - x_i^{\mathrm{def}}$, where x_i^{def} / x_i^{ref} is the corresponding measured properties (e.g. ϵ_i, w_i and so on) of the atom at the defect site and reference site, respectively. In addition, we also include $\Delta\mathrm{dip}$ and x_{sp} into this descriptor set to capture the possible high-order correlations between them and the solute-defect interaction energies. As a result, there are 23 descriptors in the set, $\boldsymbol{D} = \{D_i, D_j, \dots\}$.

b. The residual-correction function $f_{\mathrm{r-c}}(D_i, D_j, \dots)$

In the present work, we first performed covariance analysis between the regression residuals of the linear model and each of the potential descriptors to search whether there are strong and global correlations. Unfortunately, the result of the covariance analysis is negative. Therefore, instead of building a global function with analytical expression (e.g. another linear model), we attempt to use local regression methods to construct the residual-correction function based on the above set of potential descriptors. Here, the local regression methods mean that the residual-correction function behaves differently depending on the detailed values of all potential electronic descriptors ($D_i, D_j, \dots$), so this "local" feature is appropriate to deal with some outliers for the linear model prediction due to some abnormal electronic features as discussed in Supplementary Note 8.

Specifically, a statistical model is proposed and developed based on a sophisticated local regression model implemented in the Locfit package[22–25]. The model performs a series of weighted multivariate linear regressions within a moving window across the descriptor space, with a kernel-based weight function that gives the greatest weight to observations near the center of the window, producing a smooth curve that runs through the middle of the observations[23–25]. Within a cross-validation framework, the developed statistical model: (i) searches the descriptors that provide best regression accuracy on average in all five solute-matrix systems studied in the present work; and (ii) establishes a residual-correction function by recording the corresponding regression models. In the model, there are three major steps as described in the following.

First, for each of the studied solute-matrix systems, we take the residuals (Δ_{linear}) of the linear model, where $\Delta_{\text{linear}} \equiv E_{\text{int}}^{\text{fix}}(\text{DFT}) - (a_1\Delta\text{dip} + a_2 x_{\text{sp}})$, as the response to perform multivariate local linear regressions using the Locfit model[22]. The regression variables are taken from the set of descriptors discussed above, $\boldsymbol{D} = \{D_i, D_j, \ldots\}$. To reduce the risk of overfitting, we limited the dimension of the variable space to be no larger than four, meaning that at a time the local regression is only run with a subset of descriptors, d_n, where $d_n \subseteq \boldsymbol{D}$ and the size of $d_n \leq 4$. It is found that there is no significant improvement on the regression accuracy for the model with more than four descriptors. Therefore, the total number of d_n that can be used for regression is 10902 ($\text{C}_{23}^4 + \text{C}_{23}^3 + \text{C}_{23}^2 + \text{C}_{23}^1 = 10902$). To evaluate the predictive ability of each d_n, the corresponding Locfit regression is performed in a leave-one-out cross-validation (LOOCV) scenario. Specifically, a k-fold cross-validation is performed, where k is the number of data in Δ_{linear}. At each fold, one data point is removed out from Δ_{linear} to use as a test set to validate the prediction from the regression model that is trained with the rest of the $k - 1$ data points in Δ_{linear}. The prediction error of each fold, ε_l, is calculated as, $\varepsilon_l = 1/2(\text{RMSE}_{\text{training}} + \text{RMSE}_{\text{testing}})$, due to a relatively small data size of Δ_{linear}. This regression-validation process is repeated k times until all the data in Δ_{linear} have been used as the test data once. Then, the overall prediction error of the LOOCV process is calculated as, $\varepsilon = \frac{1}{k}\sum_{l=1}^{k}\varepsilon_l$. The d_n set with a smaller ε is considered to have a stronger predictive ability. Overall, this cross-validation evaluation is performed for each of the d_n set in regard to the Δ_{linear} from each of the solute-matrix systems studied in the present work (i.e. W-Ta, W-Re, W-Pt, Ta-Hf and Ta-Os).

Second, based on the results of the cross-validation evaluation, we propose a method to search the descriptor subsets (d_n) that have strong predictive abilities on average in all the five solute-matrix systems. Ideally, there should be just one set of independent descriptors, which yields the lowest prediction error in all the five solute-matrix systems if the size of the training data is large enough, since a general correlation should be conserved between electronic descriptors and the solute-defect interaction energies. However, in practice, for each of the solute-matrix system, we found that there are several d_n of which the prediction errors from the cross-validation evaluation are low

and close to each other. This is because the size of training data is relatively small and the descriptors in $\boldsymbol{D} = \{D_i, D_j, \dots\}$ are not rigorously independent with each other (the covariance is not close to zero). In addition, it is found that the descriptor subset that yields the lowest prediction error in one solute-matrix system will also yield low prediction error in other systems, but not the lowest one. On the one hand, these results imply that there are indeed strong correlations between Δ_{linear} and some certain electronic descriptors in $\boldsymbol{D} = \{D_i, D_j, \dots\}$. On the other hand, because of the limitations on the training data size and the descriptor constructions in the present work, we have not found the "ideal" subset of the descriptors that yields the lowest prediction error in all the five solute-matrix systems. Therefore, as an alternative solution, here we propose a method to rank all the tested descriptor subsets (d_n) and search the subsets that have strong predictive abilities on average in all the five solute-matrix systems, described as follows.

For each of the tested d_n, based on its prediction error ($\varepsilon_{d_n}^X$) from the LOOCV evaluation, we introduce a factor, $\gamma_{d_n}^X$, to quantitively represent the predictive ability of d_n in a specific solute-matrix system, X, which is,

$$\gamma_{d_n}^X = \frac{\varepsilon_{d_n}^X - \alpha^X}{\beta^X - \alpha^X} \tag{8}$$

where,

$$\alpha^X = \min\{\varepsilon_{d_1}^X, \varepsilon_{d_2}^X, \varepsilon_{d_3}^X, \dots \varepsilon_{d_n}^X, \dots .\} \tag{9}$$

$$\beta^X = \max\{\varepsilon_{d_1}^X, \varepsilon_{d_2}^X, \varepsilon_{d_3}^X, \dots \varepsilon_{d_n}^X, \dots .\} \tag{10}$$

X stands for the solute-matrix system of W-Ta, W-Re, W-Pt , Ta-Hf or Ta-Os, respectively.

Based on Supplementary Equation 8 above, $\gamma_{d_n}^X$ will have a value between 0 and 1 in each solute-matrix system X. The descriptor subset that yields the smallest prediction error has a $\gamma_{d_n}^X$ equal to 0, while the one yields the largest prediction error has a $\gamma_{d_n}^X$ equal to 1. The descriptor subset with a smaller $\gamma_{d_n}^X$ means that it has a stronger predictive ability in the solute-matrix system, X.

Based on $\gamma_{d_n}^X$, we evaluate the average predictive ability of each d_n in all the five solute-matrix systems. Here, we consider that a d_n will have an averagely strong predictive ability if both the mean and standard deviation of its $\gamma_{d_n}^X$ over all the five solute-matrix systems are small. Accordingly, we introduce another factor, Γ_{d_n}, for quantitative evaluation, which is calculated as,

$$\Gamma_{d_n} = \frac{M_{d_n} - M_{\min}}{M_{\max} - M_{\min}} + \frac{S_{d_n} - S_{\min}}{S_{\max} - S_{\min}} \tag{11}$$

where,

$$M_{d_n} = \frac{1}{5} \sum_X \gamma_{d_n}^X \tag{12}$$

$$S_{d_n} = \sqrt{\frac{\sum_X \left(\gamma_{d_n}^X - M_{d_n}\right)^2}{5}} \tag{13}$$

$$M_{\min/\max} = \min/\max\{M_{d_1}, M_{d_2}, \ldots M_{d_n}, \ldots.\} \tag{14}$$

$$S_{\min/\max} = \min/\max\{S_{d_1}, S_{d_2}, \ldots S_{d_n}, \ldots.\} \tag{15}$$

Here X stands for W-Ta, W-Re, W-Pt, Ta-Hf or Ta-Os, respectively. Based on Supplementary Equation 11 above, a d_n with a smaller Γ_{d_n} will generally have a stronger predictive ability in all the five solute-matrix systems. Moreover, we can rank all the d_n according to the magnitude of Γ_{d_n}, from the smallest to the largest. Supplementary Figure 16 shows the values of the smallest thirty Γ_{d_n}.

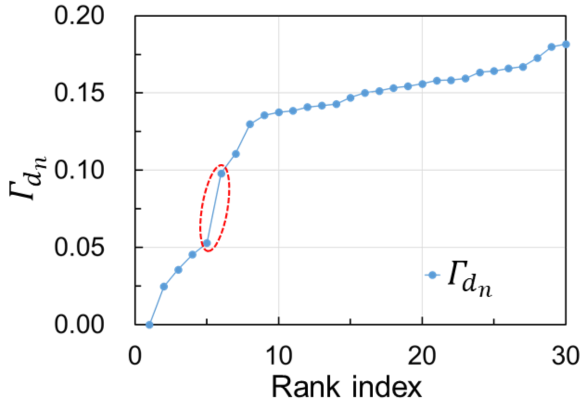

Supplementary Figure 16. The values of the smallest thirty Γ_{d_n} that calculated based on Supplementary Equation 11 among all the tested descriptor subsets, d_n.

As shown by the red circle in Supplementary Figure 16, there is a significant increase in the values of Γ_{d_n} between the 5th and 6th rank index. This observation strongly suggests that, among all the tested d_n, there are five of them that have much stronger predictive ability in the solute-matrix systems studied in the present work. The descriptor information of these five subsets are shown in Supplementary Table 7 below.

Supplementary Table 7. The top five descriptor subsets based on the rank of the smallest Γ_{d_n}.

Rank	Descriptors
1	Bimodality of the entire *d*-orbitals, Δdip;<br>Bimodality of the e_g orbital set, $\Delta dip(e_g)$;<br>Center of the e_g orbital set, ϵ_{e_g};<br>Center of the entire *sp*-orbital, ϵ_{sp}.
2	Bimodality of the entire *d*-band, Δdip;<br>Bimodality of the e_g orbital set, $\Delta dip(e_g)$;<br>Center of the entire *d*-orbitals, ϵ_d;<br>Center of the entire *s*-orbital, ϵ_s.
3	Bimodality of the entire *d*-orbitals, Δdip;<br>Bimodality of the e_g orbital set, $\Delta dip(e_g)$;<br>Center of the t_{2g} orbital set, $\epsilon_{t_{2g}}$;<br>Center of the entire *sp*-orbital, ϵ_{sp}.
4	Bimodality of the entire *d*-band, Δdip;<br>Bimodality of the e_g orbital set, $\Delta dip(e_g)$;<br>Center of the entire *d*-orbitals, ϵ_d;<br>Center of the entire *sp*-orbital, ϵ_{sp}.
5	Bimodality of the entire *d*-orbitals, Δdip;<br>Bimodality of the e_g orbital set, $\Delta dip(e_g)$;<br>Center of the e_g orbital set, ϵ_{e_g};<br>Center of the entire *s*-orbital, ϵ_s.

Third**,** we construct the residual-correction function, $f_{\mathrm{r-c}}(D_i, D_j, \ldots)$, based on the descriptor subsets in Supplementary Table 7 above, as these subsets show much stronger predictive abilities on Δ_{linear} of all the studied solute-matrix systems compared to other subsets. Specifically, for each of the solute-matrix systems, we utilize all the data in the corresponding Δ_{linear} to perform the Locfit-based local regression[22,23] using each of the subset in Supplementary Table 7 as regression variables. The corresponding regression function, η_i, is recorded. Then, the residual-

correction function, $f_{\mathrm{r-c}}(D_i, D_j, \ldots)$, is taken as the average value of the predictions from the five η_i,

$$f_{\mathrm{r-c}}(D_i, D_j, \ldots) = \frac{1}{5}\sum_{i=1}^{5} \eta_i \tag{16}$$

where $\eta_i = \mathrm{Locfit}(\Delta_{\mathrm{linear}}, d_i)$ based on one of the five descriptor subsets d_i listed in Supplementary Table 7.

Supplementary Figure 17

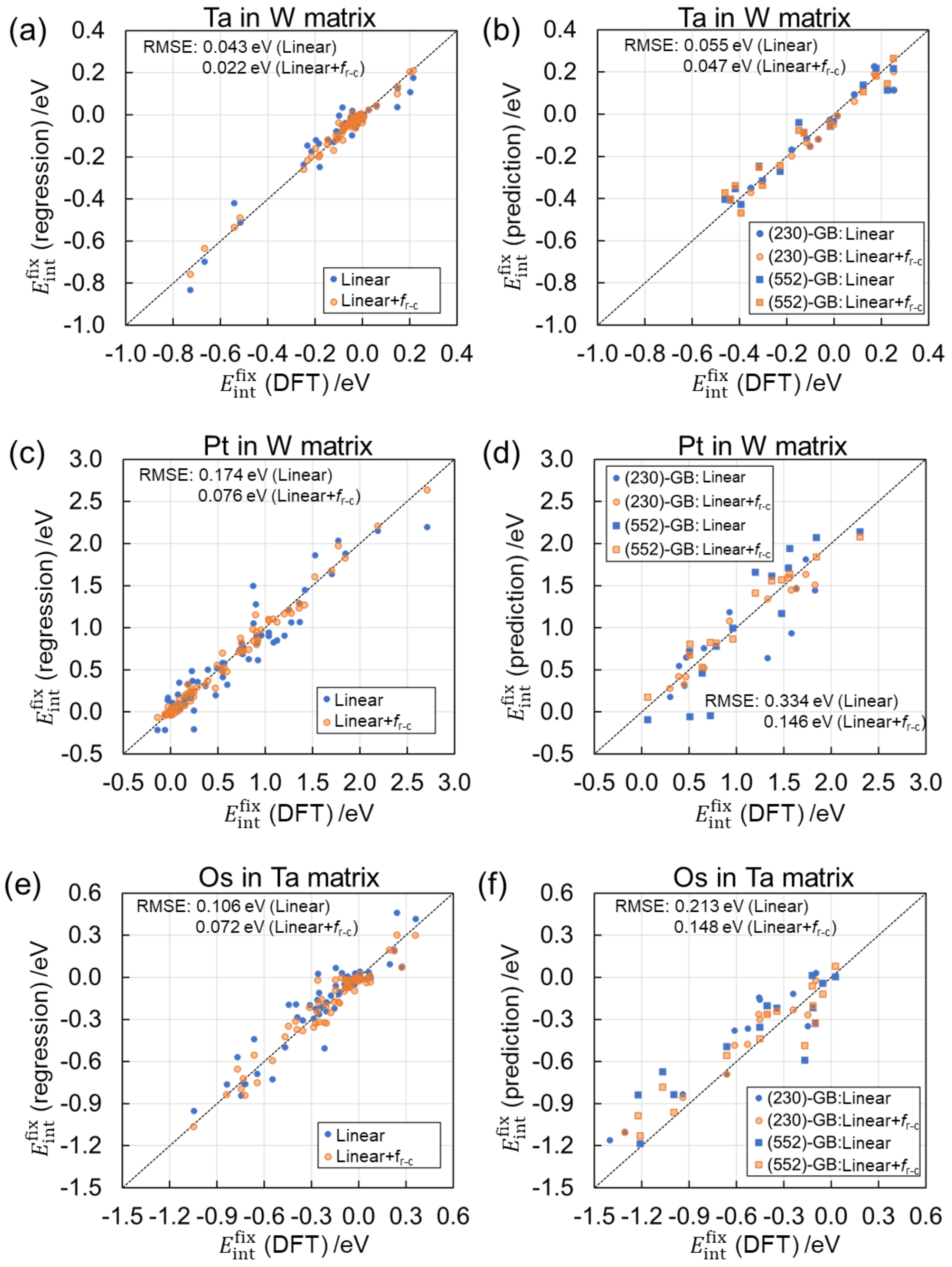

Supplementary Figure 17. Comparison between the E_{int}^{fix} from DFT calculations and from the linear model (blue) based on Equation (1) in the main text and the linear+$f_{r\text{-}c}$ model (orange) based on Equation (4) in the main text. (a) and (b): W-Ta system; (c) and (d): W-Pt system; (e) and (f): Ta-Os system. (a), (c) and (e): Regression results based on the dataset in Figs. 3 and 4 in the main text; (b), (d) and (f): Results of the Σ13 (230) GB and Σ27 (552) GB to test the predictive ability of the linear model (blue) and the linear+$f_{r\text{-}c}$ model (orange).

Supplementary Figure 18

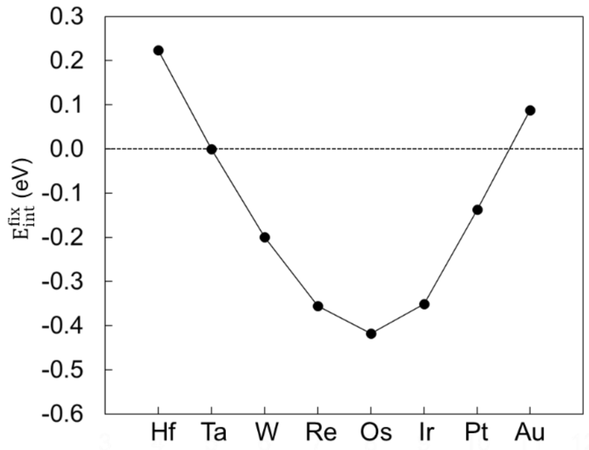

Supplementary Figure 18. Calculated interaction energies of the $(1\bar{1}0)[111]$ GSF with the transition metal solutes in the Ta matrix. The GSF has a relative displacement distance equal to a half of the full Burgers vector. The interaction energies are calculated under the fixed-lattice condition based on optimized GSF structure in pure Ta.

Supplementary Note 10

Electronic dependence of the regression coefficient of the x_{sp} term

As shown in Table 1 in the main text, the regression coefficient of the x_{sp} term, *a2*, generally has a positive sign if the solute element has fewer *d* electrons than the matrix element (e.g. W-Ta and Ta-Hf), while a negative sign if the difference in the number of d electrons is reversed. This correlation can be understood in terms of the difference in the spatial extent of *d*-orbital between solute and matrix elements. In the present work, the interaction energy is defined as the energy difference between the defect structures with a solute atom occupying the reference site far away and a site near the defect. Therefore, the second part of Equation (1) (ΔE_{sp}) in the main text can be approximated as an energy difference written as,

$$\Delta E_{\mathrm{sp}} \approx \left(E_{\mathrm{sol\_sp}}^{\mathrm{ref}} + E_{\mathrm{mat\_sp}}^{\mathrm{def}}\right) - \left(E_{\mathrm{sol\_sp}}^{\mathrm{def}} + E_{\mathrm{mat\_sp}}^{\mathrm{ref}}\right) \quad (17)$$

where $E_{\mathrm{sol\_sp}}^{\mathrm{def}}$ and $E_{\mathrm{sol\_sp}}^{\mathrm{ref}}$ are the energy gain of the solute atom associated with the *sp-d* hybridization while $E_{\mathrm{mat\_sp}}^{\mathrm{def}}$ and $E_{\mathrm{mat\_sp}}^{\mathrm{ref}}$ are the energy gain of the matrix element atom. The superscript *def* represents the atom occupying a defect site and *ref* represents that the atom occupying the reference site. Combining Supplementary Equation 17 with Supplementary Equation 3, we have,

$$\Delta E_{\mathrm{sp}} \propto \frac{r_{\mathrm{sol}}^{\frac{3}{2}} r_{\mathrm{mat}}^{\frac{3}{2}}}{\left(V_{\mathrm{vor}}^{\mathrm{ref}}\right)^{\frac{5}{3}}} + \frac{r_{\mathrm{mat}}^{\frac{3}{2}} r_{\mathrm{mat}}^{\frac{3}{2}}}{\left(V_{\mathrm{vor}}^{\mathrm{def}}\right)^{\frac{5}{3}}} - \frac{r_{\mathrm{sol}}^{\frac{3}{2}} r_{\mathrm{mat}}^{\frac{3}{2}}}{\left(V_{\mathrm{vor}}^{\mathrm{def}}\right)^{\frac{5}{3}}} - \frac{r_{\mathrm{mat}}^{\frac{3}{2}} r_{\mathrm{mat}}^{\frac{3}{2}}}{\left(V_{\mathrm{vor}}^{\mathrm{ref}}\right)^{\frac{5}{3}}} \quad (18)$$

and Supplementary Equation 18 can be rewritten as,

$$\Delta E_{\mathrm{sp}} \propto r_{\mathrm{mat}}^{\frac{3}{2}} \cdot \left(r_{\mathrm{sol}}^{\frac{3}{2}} - r_{\mathrm{mat}}^{\frac{3}{2}}\right) \cdot \left(V_{\mathrm{vor}}^{\mathrm{ref}}\right)^{-\frac{5}{3}} \cdot \left(1 - \frac{\left(V_{\mathrm{vor}}^{\mathrm{def}}\right)^{-\frac{5}{3}}}{\left(V_{\mathrm{vor}}^{\mathrm{ref}}\right)^{-\frac{5}{3}}}\right) \quad (19)$$

Based on Supplementary Equation 4, if we assume the $\epsilon_{\mathrm{sp}}^{\mathrm{def}} / \epsilon_{\mathrm{sp}}^{\mathrm{ref}}$ term usually has a value around 1, the expression on the right side of Supplementary Equation 19 can be approximately written as $r_{\mathrm{mat}}^{\frac{3}{2}} \cdot \left(r_{\mathrm{sol}}^{\frac{3}{2}} - r_{\mathrm{mat}}^{\frac{3}{2}}\right) \cdot \left(V_{\mathrm{vor}}^{\mathrm{ref}}\right)^{-\frac{5}{3}} \cdot x_{\mathrm{sp}}$. As a result, the solute element with fewer *d* electrons should have a positive a_2 as it also has a more spread

spatial extent of *d*-orbital compared to the matrix element[18] (i.e. $r_{\mathrm{sol}} > r_{\mathrm{mat}}$), and vice versa for the solute element with more *d* electrons than the matrix element.

Supplementary References:

1. Kong, X. S. *et al.* First-principles calculations of transition metal-solute interactions with point defects in tungsten. *Acta Materialia* **66**, 172–183 (2014).

2. Bacon, D. J., Barnett, D. M. & Scattergood, R. O. Anisotropic continuum theory of lattice defects. *Progress in Materials Science* **23**, 51–262 (1980).

3. Yasi, J. A. & Trinkle, D. R. Direct calculation of the lattice Green function with arbitrary interactions for general crystals. *Physical Review E* **85**, 66706 (2012).

4. Trinkle, D. R. Lattice Green function for extended defect calculations: Computation and error estimation with long-range forces. *Physical Review B* **78**, 014110 (2008).

5. Hu, Y.-J. *et al.* Solute-induced solid-solution softening and hardening in bcc tungsten. *Acta Materialia* **141**, 304–316 (2017).

6. Wu, X. *et al.* First-principles determination of grain boundary strengthening in tungsten: Dependence on grain boundary structure and metallic radius of solute. *Acta Materialia* **120**, 315–326 (2016).

7. Zhu, Q., Samanta, A., Li, B., Rudd, R. E. & Frolov, T. Predicting phase behavior of grain boundaries with evolutionary search and machine learning. *Nature Communications* **9**, 467 (2018).

8. Frolov, T. *et al.* Grain boundary phases in bcc metals. *Nanoscale* **10**, 8253–8268 (2018).

9. Hodges, L., Ehrenreich, H. & Lang, N. D. Interpolation scheme for band structure of noble and transition metals: ferromagnetism and neutron diffraction in Ni. *Physical Review* **152**, 505 (1966).

10. Pettifor, D. G. Theory of energy bands and related properties of 4d transition metals. III. s and d contributions to the equation of state. *Journal of Physics F: Metal Physics* **8**, 219 (1978).

11. Pettifor, D. G. Theory of energy bands and related properties of 4d transition metals. I. Band parameters and their volume dependence. *Journal of Physics F: Metal Physics* **7**, 613 (1977).

12. Pettifor, D. G. A physicist's view of the energetics of transition metals. *Calphad* **1**, 305–324 (1977).

13. Mrovec, M. *et al.* Bond-order potential for simulations of extended defects in tungsten. *Physical Review B* **75**, 104119 (2007).

14. Znam, S., Nguyen-Manh, D., Pettifor, D. G. & Vitek, V. Atomistic modelling of TiAl I. Bond-order potentials with environmental dependence. *Philosophical Magazine* **83**, 415–438 (2003).

15. Mueller, F. M. Combined interpolation scheme for transition and noble metals. *Phyiscal Review* **153**, 659 (1967).

16. Pettifor, D. G. Accurate resonance-parameter approach to transition-metal band structure. *Physical Review B* **2**, 3031 (1970).

17. Lambert, R. M. & Pacchioni, G. *Chemisorption and Reactivity on Supported Clusters and Thin Films:: Towards an Understanding of Microscopic Processes in Catalysis*. **331**, (Springer Science & Business Media, 2013).

18. Xin, H., Holewinski, A., Schweitzer, N., Nikolla, E. & Linic, S. Electronic structure engineering in heterogeneous catalysis: Identifying novel alloy catalysts based on rapid screening for materials with desired electronic properties. *Topics in Catalysis* **55**, 376–390 (2012).

19. Harrison, W. A. *Electronic structure and the properties of solids: the physics of the chemical bond*. (Courier Corporation, 2012).

20. Lüthi, B., Ventelon, L., Rodney, D. & Willaime, F. Attractive interaction between interstitial solutes and screw dislocations in bcc iron from first principles. *Computational Materials Science* **148**, 21–26 (2018).

21. Wang, J., Janisch, R., Madsen, G. & Drautz, R. First-principles study of carbon segregation in bcc iron symmetrical tilt grain boundaries. *Acta Materialia* **115**, 259–268 (2016).

22. Loader, C. *Local regression and likelihood*. (Springer Science & Business Media, 2006).

23. De Jong, M. *et al.* A statistical learning framework for materials science:

application to elastic moduli of k-nary inorganic polycrystalline compounds. *Scientific Reports* **6**, 34256 (2016).

24. Cleveland, W. S. Robust locally weighted regression and smoothing scatterplots. *Journal of the American statistical association* **74**, 829–836 (1979).

25. Stone, C. J. Consistent nonparametric regression. *The annals of statistics* **5**, 595–620 (1977).
